# Anticandidal Effect and Mechanisms of Monoterpenoid, Perillyl Alcohol against *Candida albicans*

**DOI:** 10.1371/journal.pone.0162465

**Published:** 2016-09-14

**Authors:** Moiz A. Ansari, Zeeshan Fatima, Saif Hameed

**Affiliations:** Amity Institute of Biotechnology, Amity University Haryana, Gurgaon (Manesar)-122413, India; Louisiana State University, UNITED STATES

## Abstract

This study explored the antifungal potential of perillyl alcohol (PA), a natural monoterpene alcohol, against most prevalent human fungal pathogen *C*. *albicans*, its clinical isolates and four non*-albicans* species of *Candida*. To resolve the potential mechanisms, we used whole genome transcriptome analyses of PA treated *Candida* cells to examine the affected cellular circuitry of this pathogen. The transcriptome data revealed a link between calcineurin signaling and PA as among the several categories of PA responsive genes the down regulation of calcineurin signaling gene *CNB1* was noteworthy which was also confirmed by both molecular docking and susceptibility assays. We observed that PA treated *Candida* phenocopied compromised calcineurin pathway stress responses and turned sensitive to alkaline pH, ionic, membrane, salinity, endoplasmic reticulum and serum stresses. Indispensability of functional calcineurin was further confirmed as calcineurin mutant was hypersensitive to PA while constitutively expressed calcineurin strain remained resistant. We explored that PA leads to perturbed membrane integrity as depicted through depleted ergosterol levels and disrupted pH homeostasis. Moreover, PA caused cell wall damage which was evident from hypersensitivity against cell wall perturbing agents (congo red, calcoflour white), SEM and enhanced rate of cell sedimentation. Furthermore, PA inhibited potential virulence traits including morphological transition, biofilm formation and displayed diminished capacity to adhere both to the polystyrene surface and buccal epithelial cells. The study also revealed that PA leads to cell cycle arrest and mitochondrial dysfunction in *C*. *albicans*. Together, the present study provides enough evidence for further work on PA so that better strategies could be employed to treat *Candida* infections.

## Introduction

*Candida albicans* is an opportunistic fungus residing in the human body due to its commensal nature [1]. It becomes a great threat particularly in immunocompromised conditions due cancer, HIV, organ transplantation [2, 3]. The constrained armory of conventional antifungal treatments for candidiasis depends profoundly on polyenes, azoles and echinochandins, but they either have tapered therapeutic index, lower bioavailability, poor gastrointestinal absorption or stern side effects [4]. The inevitable consequence due to their prolonged usage has led to development of multi drug resistance (MDR) which is a major impediment against efficient therapeutics. Therefore, with continuously escalating global prevalence of MDR, poor efficiency of the currently applicable drugs, side effects, high costs and stagnation in development of new drugs, a question is now getting posed against effectiveness of above mentioned drugs [3, 5]. Therefore, it has become a serious challenge to explore novel drugs with newer targets against this fungal pathogen. Use of natural compounds with antifungal properties has gained prominence and substantial interest as they have lesser side effects, being economical and to our knowledge cause no resistance [6]. Moreover, naturally occurring compounds such as, phenolic compounds, essential oils, terpenoids, flavonoids are already reported to exhibit antifungal activities [7–9].

Perillyl alcohol (PA) is hydroxylated metabolite of d-limonene monocyclic monoterpene isolated from the essential oil of lavendin, peppermint, spearmint, cherries, celery seeds and several other plants [10]. PA is already approved by the U.S. Food and Drug Administration as a food additive that can be safely consumed by human displaying its non-toxic nature [11]. Anticancerous properties of PA have been extensively studied as apparent from wide range of studies [12, 13]. For instance, its in-vitro anti cancerous activity against breast cancer, in vivo intracranial triple negative tumor growth [14], pancreatic cancer [15], and metastatic colorectal cancer [16] has been well documented. Preliminary antifungal activity of PA has also been reported [17], however the precise mechanism of its action against *C*. *albicans* was elusive.

In this study, we deciphered the antifungal effect of PA not only against *C*. *albicans* but also non-*albicans* species of *Candida* with the possible underlying mechanisms. Transcriptional profiling of *C*. *albicans*, validated by RT-PCR, along with several biochemical analyses confirmed that PA employs multiple mechanisms to inhibit *Candida* growth. This is the first study reporting antifungal mechanism of PA against *C*. *albicans* which will widen the resources of potential antifungal agents and lay foundations for new therapeutics.

## Materials and Methods

All Media chemicals YEPD (Yeast Extract Peptone Dextrose), nutrient broth, yeast nitrogen base w/o amino acid and ammonium sulphate (YNB w/o amino acid and ammonium sulphate), agar, rhodamine 6G (R6G), 2-deoxy glucose (2-DOG), horse serum, 2,4 dinitrophenol (2,4 DNP), n- heptane, formamide, osmium tetroxide (OsO_4_), hexamethyldisilizane (HMDS), glutaraldehyde, propidium iodide were purchased from Himedia (Mumbai, India). Sodium chloride (NaCl), calcium chloride (CaCl_2_), lithium chloride (LiCl), potassium chloride (KCl), mannitol, di-sodium hydrogen orthophosphate, potassium di-hydrogen orthophosphate, di-potassium hydrogen orthophosphate, sodium hydroxide, D-glucose, sodium dodecyl sulphate (SDS), potassium hydroxide (KOH), ammonium sulphate ((NH_4_)_2_SO_4_), dimethyl sulphoxide (DMSO) were obtained from Fischer Scientific. Tetrazolium salt 3-[4, 5-dimethylthiazol-2-yl]-2, 5-diphenyltetrazolium bromide (MTT) was purchased from SRL, Mumbai. Calcofluor white (CFW), congo red (CR), perillyl alcohol (PA), diethyl pyrocarbonate (DEPC), 4-morpholinepropanesulfonic acid (MOPS), tri-reagent, DNase were obtained from Sigma Chemical Co. (St. Louis, MO, USA).

### Growth media and strains

All the strains (Table 1) of *C*. *albicans* were cultured in YEPD broth with the composition of yeast extract 1% (w/v), peptone 2% (w/v) and dextrose 2% (w/v). For agar plates 2% (w/v) agar was added to the media. YPG agar plate for non fermentable source of carbon was made with the composition of yeast extract 1% (w/v), peptone 2% (w/v), glycerol 2% (v/v) and 2% (w/v) agar. All *Candida* strains were stored in 30% (v/v) glycerol stock at -80°C. The cells were freshly revived on YEPD broth and transferred to agar plate. The cells were grown at 30°C on agar plate before each study to ensure revival of the strains.

**Table 1:** List of strains used.

Name	Strain	Genome	Reference
*C*. *albicans*	SC5314	Reference strain	[18]
*C*. *albicans*	D1	Clinical isolate*	Safdarjung Hospital
*C*. *albicans*	D2	Clinical isolate*	Safdarjung Hospital
*C*. *albicans*	D4	Clinical isolate*	Safdarjung Hospital
*C*. *albicans*	D7	Clinical isolate*	Safdarjung Hospital
*C*. *albicans*	D18	Clinical isolate*	Safdarjung Hospital
*Candida tropicalis*	D9	Clinical isolate*	Safdarjung Hospital
*Candida parapsilosis*	D11	Clinical isolate*	Safdarjung Hospital
*Candida krusei*	D46	Clinical isolate*	Safdarjung Hospital
*Candida glabrata*	D10	Clinical isolate*	Safdarjung Hospital
*C*. *albicans*	DAY185	URA3/ura3Δ::λimm434 HIS1/his1::hisG ARG4/arg4::hisG	[19]
*C*. *albicans (Δcrz1/Δcrz1)*	OCC1.1	ura3Δ::λimm434/ura3Δ::λimm434his1::hisG::HIS1/his1::hisGarg4::hisG/arg4::hisG crz1::UAU1/crz1::ARG4	[19]
*C*. *albicans (Δcnb1/Δcnb1)*	JRB64	ura3Δ::λimm434/ura3Δ::λimm434his1::hisG::HIS1/his1::hisGarg4::hisG/arg4::hisGcnb1::UAU1/cnb1::ARG4	[19]
*C*. *albicans (CNB1-1/CNB1)*	YAG237	CNB1 mutant having hyperactive allele	[18]

*The clinical isolates were derived from diabetic patients having oral candidiasis.

### Drug susceptibility testing

Drug susceptibility was tested using minimum inhibitory concentration (MIC) and by spot assays as described below:

#### Spot assay

Spot assays for the strains were determined using a known method as described elsewhere [8, 9]. Briefly, for the spot assay, 5μl of fivefold serially diluted yeast cultures (cells suspended in normal saline to an OD_600_ nm of 0.1) were spotted onto YEPD plates in the absence (control) and presence of the drugs. Growth was not affected by the presence of solvent used in the examination (data not shown). Growth difference was measured after incubation for 48 hours at 30°C. The concentrations used in this study are specified in figure legends.

#### Minimum Inhibitory Concentration (MIC)

MIC was determined by broth dilution method as described in method M27-A3 from the Clinical and Laboratory Standards Institute (CLSI) formerly NCCLS (National Committee for Clinical Laboratory Standards) [20]. Briefly, 100μl of media was placed in each well of 96 wells plate following addition of the drug and the remaining media (to make final volume as 200μl in first well) and then was serially diluted. 100μl of cell suspension (in normal saline to an OD_600_ 0.1) was added to each well and OD_600_ was measured after 48 hours at 30°C. The MIC_80_ was defined as the concentration at which 80% of the growth was inhibited.

### RNA Isolation

Isolation of RNA from *C*. *albicans* was carried out by using combination of Trizol and Qiagen RNeasy mini kit with DNase treatment [8]. The cells were diluted into 50 ml fresh YEPD broth at OD_600_ of 0.1 (10^6^ cells ml^-1^) in absence and presence of PA (175μg ml^-1^), and grown at 30°C till OD_600_ of 1.0. Cells were harvested by transferring the cells into a centrifuge tube. Three separate experimental replicate cultures of each condition were used. Lysate was prepared by Trizol, chloroform was added to lysate and samples were subject to centrifugation. The supernatant was taken from previous step and after adding ethanol to the supernatant, the mixture was loaded on Qiagen column and Manufacturer’s guidelines were followed from DNase treatment step. Purity (Ratio of 260/280 and 260/230) and concentration was assessed by NanoDrop 1000. Integrity of RNA was assessed on Agilent Bioanalyzer 2100.

### cDNA synthesis and hybridization

For RT-PCR, cDNA was synthesized with the cDNA synthesis kit RevertAid H Minus kit (Invitrogen) from the RNA isolated by trizole method [8]. The samples for gene expression were labeled using Agilent Quick-Amp labeling Kit (p/n5190-0442). 500ng each of total RNA were reverse transcribed at 40°C using oligo dT primer tagged to a T7 polymerase promoter and converted to double stranded cDNA. Synthesized double stranded cDNA were used as template for cRNA generation. cRNA was generated by in vitro transcription and the dye Cy3 CTP(Agilent) was incorporated during this step. The cDNA synthesis and in vitro transcription steps were carried out at 40°C. Labeled cRNA was cleaned up using Qiagen RNeasy columns and quality assessed for yields and specific activity using the Nanodrop ND-1000. 1000ng of labeled cRNA sample were fragmented at 60°C and hybridized on to a Genotypic designed *Candida albicans*_8x15K (AMADID: 026377). Fragmentation of labeled cRNA and hybridization were done using the Gene Expression Hybridization kit (Agilent Technologies, In situ Hybridization kit, Part Number 5190–0404). Hybridization was carried out in Agilent’s Surehyb Chambers at 65°C for 16 hours.

### Scanning and data analysis

The hybridized slides were washed using Agilent Gene Expression wash buffers (Agilent Technologies, Part Number 5188–5327) and scanned using the Agilent Microarray Scanner (Agilent Technologies, Part Number G2600D). Data extraction from images was done using Feature Extraction software Version 11.5.1.1 of Agilent. Images were quantified using Feature Extraction Software (Version-11.5, Agilent). Feature extracted raw data was analyzed using GeneSpring GX Version 12.0 software from Agilent. Normalization of the raw data was done in GeneSpring GX using Quantile method (Quantile normalization makes the distribution of expression values of all samples similar in an experiment). After, normalization all statistical parameters of the sample i.e., mean, median and percentile of all samples will be identical. It works well with reducing variance between arrays. Differential expression patterns were identified among the samples. Significant genes up regulated fold > 1.5 (log_2_) and down regulated < -1.5 (log_2_) in the PA treated samples with respect to control sample were identified. Statistical student T-test and *P* value among the replicates was calculated based on volcano plot algorithm. Differentially regulated genes were clustered using hierarchical clustering based on Pearson coefficient correlation algorithm to identify significant gene expression patterns. The Significant Functional classification of differentially regulated genes was performed using GeneSpring GX software gene ontology.

### Microarray data gene accession number

Microarray data used in this study is fully described in GEO and the raw as well as normalized data files have been deposited under accession number GSE76383.

### RT-PCR

For validation of the microarray results, reverse transcriptase (RT) PCR was done as described in the RevertAid H Minus kit (Invitrogen) [8]. Briefly, 5μg isolated RNA was DNase treated at 37°C for 30 min and reaction was terminated by adding 1μl of 25mM EDTA and incubated at 65°C for 60 min. RNA was subsequently primed with oligo (dT)_18_ for cDNA synthesis at 42°C for 60 min. Reverse transcription reaction was terminated by heating at 70°C for 5 min. The synthesized cDNA product (2μl) was directly used for PCR amplification reaction (50 μl) using gene specific forward and reverse primers (S1 Table). The amplified products were gel electrophoresed and the densities of bands (for genes of interest) were measured and quantified by normalizing to that of the constitutively expressed actin gene (*ACT1*).

### Susceptibility assays

Susceptibility was assessed using spot assays as described above. The following stock solutions were used (the solvents used are given in parenthesis): SDS, 10% w/v (water); NaCl, 5M (water), LiCl 5M (water), CaCl_2_ 5M (water), DTT 1M (water), FLC 5mgml^-1^ (water), CFW 1mgml^-1^, CR 5mgml^-1^. Cells were spotted on to YEPD plates in the absence (control) and presence of the PA at its sub inhibitory concentration (175 μgml^-1^) along with the chemicals at the following concentrations: SDS (0.02% w/v), NaCl (1M), LiCl (0.4M) and CaCl_2_ (0.3M), DTT (20mM), Serum (50% v/v), FLC 1μgml^-1^, CFW 10μgml^-1^, CR 10μgml^-1^. For alkaline pH 10.0, YEPD plates buffered with 155mM of Tris-HCl at pH 10 were used. Growth differences were recorded following incubation of the plates for 48 hours at 30°C.

### Molecular docking

Molecular docking study using the ligand molecules with calcineurin (PDB id: 1AUI) was conducted using Autodock 4.2 and Auto Dock Tools (ADT) v 1.5.4 from the Scripps Research Institute [21]. The ligands were set to explore and flexible to rotate most probable binding poses, while receptor was kept rigid. The grid maps representing the center of active site pocket for the ligand were calculated with Autogrid. The dimensions of the grid for calcineurin was 66 × 72 × 66 grid points with a spacing of 0.911 Å between the grid points but centered on the ligand for receptor (21.725, 4.621 and 4.136 coordinates). The present docking study was performed by each run with population of 150 individuals, rate of gene mutation 0.02, cross-over rate 0.8 and the remaining parameters were set as default. Ten poses docking conformations were generated and the best docked conformation was selected based on the Autodock binding energy (Kcal/mol), for further analysis. Finally, the results generated were visualized by PyMOL viewer for analysis minimum binding energy (Kcal/mol), Ki (Inhibition constant) value (μM), and hydrogen and hydrophobic interaction of the docked inhibitor to the modeled structure.

### Quantitation of ergosterol

Sterols were extracted by the alcoholic KOH method and the percentage of ergosterol was calculated as described previously [8, 9]. Briefly, a single *C*. *albicans* colony from an overnight YEPD agar plate culture was used to inoculate 50 ml of YEPD in presence and absence of PA (conc). Both ergosterol and 24(28)-DHE absorb at 281.5 nm, whereas only 24(28)-DHE absorbs at 230 nm. Ergosterol content is determined by subtracting the amount of 24(28)-DHE (calculated from the OD_230_) from the total ergosterol plus 24(28)-DHE content (calculated from the OD_281.5_). Ergosterol content was calculated as a percentage of the wet weight of the cells with the following equations: % Ergosterol + % 24(28)-DHE = [(A_281.5_/290) × F] / pellet weight; % 24(28)-DHE = [(A_230_/518) × F] / pellet weight and % Ergosterol = [% ergosterol + % 24(28) DHE]—% 24(28) DHE, where *F* is the factor for dilution in petroleum ether and 290 and 518 are the *E* values (in percent per centimeter) determined for crystalline ergosterol and 24(28)-DHE, respectively.

### Intracellular pH (pHi)

Intracellular pH was measured as described earlier with slight modifications [8, 9, 22, 23]. Mid-log phase cells grown in YEPD medium were harvested and washed twice with distilled water. Cells (0.1 g) were suspended in 5 ml solution containing 0.1 M KCl and 0.1 mM CaCl_2_. Desired concentration of PA (MIC_80_) was added to the suspension and pH was adjusted to 7.0 in each case. Following incubation for 30 min at 37°C with constant shaking, pH was again adjusted to 7.0. Nystatin (20 mM) dissolved in 10% DMSO was added to the unbuffered cell suspension and incubated at 37°C for 1h. The change in pH of suspension was followed on pH meter with constant stirring. The value of external pH at which nystatin permeabilization induced no further shift was taken as an estimate of pHi. Nystatin binds with sterols of plasma membrane which develops pores and allows the equilibration of protons and other ions across the membrane. Since nystatin does not affect mitochondria hence give pHi values nearer to the factual cytoplasmic pH [23].

### Relative Sedimentation

Cell sedimentation was measured spectrophotometrically as described previously with slight modifications [24]. Briefly, overnight culture of *C*. *albicans* were inoculated to 0.1 OD_600_ to both control and PA treated cells and allowed to grow till the OD_600_ reaches 1.0. The OD_600_ from each untreated and treated cultures were measured at each min till 30 minutes. Sedimentation rates were measured by recording the differences in growth from zero time point to 30 min per unit time interval and calculated as described in figure legends.

### Electron Microscopy

Damage to the cells treated with PA at its MIC_80_ value was observed by using Scanning Electron Microscopy (SEM) (Zeiss EVOMA10) [8]. The cells (~10^6^ cells) were administered to the media with and without PA and were incubated for 24h at 30°C. Sample preparation and analysis was performed by using the method as described elsewhere [8, 9]. Briefly, all cells were fixed with 2% glutaraldehyde in 0.1% phosphate buffer for 1 h at room temperature (20°C). Washed with 0.1 M phosphate buffer (pH 7.2) and post-fixed 1% OsO_4_ in 0.1 M phosphate buffer for 1 h at 4°C. The cells were dehydrated in acetone and dropped on round glass cover slip with hexamethyldisilizane (HMDS) and dried at room temperature, then sputter coating with gold and observed under the SEM (Zeiss EVOMA10) at 30K magnification for cell wall and 1000X magnification for biofilm.

### Yeast to hyphal transition

Studies of hyphal induction on *C*. *albicans* were carried out on both liquid and solid hyphal induction media. For morphological switching the growth media used were YEPD, serum (10% (v/v) serum in YEPD), spider media (1% mannitol, 0.4% K_2_HPO_4_, 1% Nutrient Broth) and SLAD (0.17g YNB w/o amino acid and ammonium sulphate; 2g glucose; 0.5ml of 10mM (NH4)_2_SO_4_). The dimorphic switching was performed using the protocol described elsewhere [8, 9]. Briefly, the culture was grown overnight at 30°C in YEPD broth before each study. The cells were harvested by centrifugation at 5000xg for 3 minutes and washed twice and incubated at 37°C for 6h with PBS to induce starvation. After incubation the cells were transferred to the indicated media in the absence (control) and presence of PA for hyphal induction and hyphae were observed under microscope.

### Biofilm formation and cell adhesion

*Candida* biofilms were checked on polystyrene surface of 96-well plates [8, 9]. An overnight culture was prepared and cell suspension of 1 x 10^7^ cells ml^-1^ was made in PBS and 100μL was inoculated in each well. The plates were incubated at 37°C for 90 min to adhere the cells on the surface. The wells were gently washed 2–3 times with PBS after 90 min to remove the non-adhered cells. The biofilm was formed by suspending 200μL of YEPD medium along with sub-inhibitory concentrations of PA (175μg ml^-1^) and one control without PA to each well of adhered cells to polystyrene 96 well plates and the plates were incubated at 37°C for 24h. After incubation, wells were washed to remove any planktonic cells and biofilms were observed under light microscope. To quantify the biofilm on the polystyrene surface of 96 well plate tetrazolium salt 3-[4, 5-dimethylthiazol-2-yl]-2, 5-diphenyltetrazolium bromide (MTT) was added by 50μl (stock solution containing 5mg/ml, diluted 1:5 in prewarmed 0.15M PBS prior to addition) in each well. The plates were incubated for 5 h at 37°C. Dimethyl sulfoxide (200μL) was added to each well to solubilize MTT formazan product and optical density was measured at 450nm. The metabolic activity of biofilm formation was calculated in percentage by comparing the drug free control with the treated cells. For cell adhesion assay same protocol was followed except that primarily treated and non treated cells were grown till OD_600_ 1.0 and after washing the adhered cells were directly quantified through MTT assay without forming biofilm.

### Adherence to epithelial cells

Adherence assay was performed as described earlier [8, 25]. Yeast cells were grown on YEPD for 24 h at 37°C and re-suspended in 2 ml of sterile PBS (pH 6.8) and washed twice by centrifugation (3000 x g, 5 min). Author voluntarily donated the epithelial cells via soft scraping of the cheek mucous membrane with sterile cotton swabs, gently stirred and washed with PBS by centrifugation (3000 x g, 5 min each). Adherence assays were developed by mixing 1 ml of each suspension in a test tube and incubated in the presence of PA at 37°C under gentle stirring for 2 h. After incubation, 0.4% of trypan blue solution was added to each tube and the mixture was gently shaken. Stained suspensions were examined under light microscopy at 40X magnification.

### Flow cytometry

The effect of PA on *C*. *albicans* cell cycle was studied as described elsewhere [8, 26] with few modifications. Briefly, overnight culture of *C*. *albicans* were inoculated to 0.1 OD_600_ to both control and sub-inhibitory concentration of PA and allowed to grow till the OD_600_ reaches 1.0. The cells were then harvested and fixed in 1ml of 70% ethanol. Fixed cells were stored at 4°C. Ethanol fixed cells were stained with propidium iodide (50μg ml^-1^) and cell cycle was analyzed using Fluorescence Activated Cell Sorter (FACS) (BD LSR-II). 20,000 events were counted and fluorescence intensity was compared between control and PA treated cells and analyzed with BD FACS Diva 6.1.3 software.

### Mitochondrial activity

Mitochondrial activity was evaluated by MTT assay as described earlier [8, 27, 28]. Overnight culture of *C*. *albicans* cells were diluted in fresh YEPD medium to an initial OD_600_ of 0.1 and grown at 30°C for 5 h. The cultures were then treated with sub-inhibitory concentration of PA (175μg ml^-1^) for 3 h. 500 μl of cells were harvested, washed twice with YEPD medium, mixed with 500μl of MTT (100μg ml^-1^, diluted in PBS) and statically incubated for further 2 h. Cells were again harvested and washed twice with YEPD medium. The pellets were then suspended in 1 ml of DMSO, incubated at 30°C with shaking for 5 min. The suspensions were centrifuged and OD_570_ of the supernatants was determined.

### Statistical analysis

All experiments were performed in triplicates (n = 3). The results are reported as mean ± standard deviation (SD) and analyzed using Student’s t test where in *P* < 0.05 was considered as statistically significant.

## Results and Discussion

### In vitro antifungal activity of PA against *C*. *albicans* and non-*albicans* species

**Fig 1:**
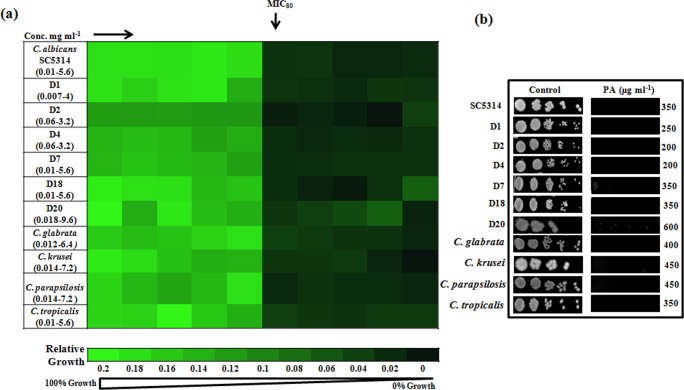
Drug susceptibility assays against *C*. *albicans* and non-*albicans* species of *Candida* in the presence of PA. **(a)** Broth microdilution assay to determine the MIC_80_ of *C*. *albicans* reference strain SC5314, clinical isolates (D1, D2, D4, D7, D18 and D20) and *C*. *glabrata*, *C*. *krusei*, *C*. *parapsilosis* and *C*. *tropicalis* in presence of PA. Data was quantitatively displayed with color (see color bar), where each shade of color represents relative optical densities of the cell. The minimum drug concentration that inhibits growth by 80% relative to the drug-free growth control is indicated as MIC_80_ for each strain. **(b)** Spot assay of *C*. *albicans* reference strain SC5314, clinical isolates (D1, D2, D4, D7, D18 and D20) and *C*. *glabrata*, *C*. *krusei*, *C*. *parapsilosis* and *C*. *tropicalis* in the absence (control) and presence of PA.

To check the efficacy of PA against *C*. *albicans*, drug susceptibility tests were performed by two independent methods viz. broth microdilution and spot assay_._ Both the assays confirmed that PA is capable to inhibit the growth of *C*. *albicans* at350 μgml^-1^; however we observed that PA was fungistatic to *C*. *albicans* at its MIC_80_ concentration. Inhibitory concentration of *C*. *albicans* was firstly evaluated by determining MIC_80_ as depicted in Fig 1A. To confirm the MIC_80_ result, spot assay was performed which corresponds with the MIC_80_ result (Fig 1B). The antifungal activity of PA was further assessed against six clinical isolates of *C*. *albicans* and we found that all the strains displayed susceptibility to PA ranging from 200–600 μg ml^-1^ (Fig 1).

We extended our investigation to assess the effect of PA on the growth of other *Candida* spp. For this also, we performed both the drug susceptibility methods with PA on different non-*albicans* species of *Candida*. The broad repertoire for antifungal activity of PA was apparent from the fact that it was also effective against other tested non-*albicans* species of *Candida* (Fig 1). The range of sensitivity for non-*albicans* species towards PA was however variable and in the following order: *C*. *tropicalis* (200 μg ml^-1^)>*C*. *glabrata* (400 μg ml^-1^)>*C*. krusei/C. *parapsilosis* (450 μg ml^-1^). Thus, the drug susceptibility testing results indicated PA to be inhibitory not only against reference and clinical isolates of *C*. *albicans* but also non-*albicans* species. Furthermore, growth curve experiment confirmed that both 175 μg ml^-1^ and 225μg ml^-1^ were subinhibitory concentrations at which PA showed partial inhibitory effect on *C*. *albicans* (S1 Fig). Thus for transcriptional profiling and subsequent biochemical and phenotypic studies, we used PA at its subinhibitory concentrations of 175μg ml^-1^ or 225μg ml^-1^ (former being preferred in most experiments due to its lower value).

### Genomewide response of *C*. *albicans* transcriptome to PA

Over expression of the drug efflux pumps of ABC major superfamily is one of the leading mechanisms responsible for development of drug resistance in *Candida* against the antifungal drugs [29, 30]. Considering the above fact we assumed that the observed antifungal activity of PA may be the result of abrogated drug efflux. Therefore, we firstly investigated the efflux of R6G, a known substrate of ABC superfamily efflux pumps, in presence of PA. Our results (S2 Fig) showed no significant difference in the efflux rates of R6G. Thus, contribution of compromised efflux pump activity in susceptibility of *Candida* cells to PA was excluded suggesting that PA acts against *Candida* via some other mechanism.

**Fig 2:**
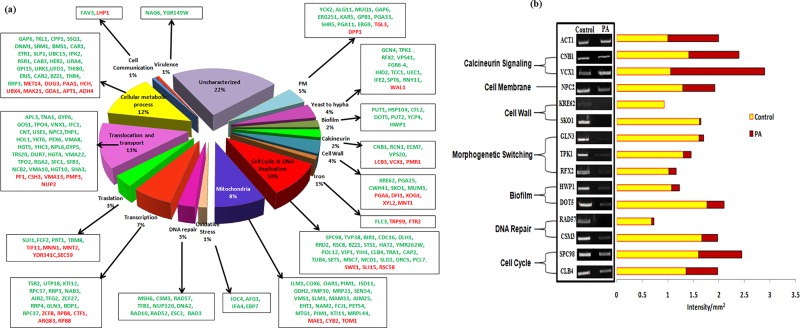
**(a) Transcriptional profiling of *C*. *albicans* in response to PA.** Pie chart showing various gene categories differentially regulated in PA treated cells. Green color show downregulated genes and red color shows upregulated genes. **(b) RT-PCR of differentially regulated genes in response to PA.** The left panels show transcript levels of *CNB1*, *VCX1*, *NPC2*, *KRE62*, *SKO1*, *GLN3*, *TPK1*, *RFX2*, *HWP1*, *DOT5*, *RAD57*, *CSM3*, *SPC98*, *CLB4* in lanes (1) Control, (2) PA (175μg ml^-1^). The right panel shows the quantitation (density expressed as Intensity/mm^2^) of the respective transcripts normalized with constitutively expressed *ACT1* transcripts.

For deeper insights into the possible mechanisms of PA against *C*. *albicans*, transcriptional profiling was performed. The microarray displayed 289 genes to be differentially regulated in PA treated cells grouped under various categories (Fig 2A). Of 289 genes, 223 genes were downregulated while the remaining 66 were upregulated in response to PA. The details of gene categories, their description and the mean log_2_ fold changes are listed in S2 and S3 Tables. Apart from the variety of genes that were differentially regulated in response to PA we could detect some significant changes in genes associated to affect antifungal drug susceptibilities. Most strikingly, we explored a link with cellular stress coping calcineurin signaling pathway (described below). The transcriptome data further unravels that PA suppresses *C*. *albicans* growth via numerous targets such as disrupting the cell wall, cell membrane, inhibiting morphogenetic switching, cell adhesion and biofilm formation, mitochondria activity, cell cycle and DNA repair. The microarray data was validated by performing RT-PCR under growth condition similar to that used for microarray and selecting 14 genes from various differentially regulated categories. The genes included calcineurin signaling (*CNB1*, *VCX1*), cell wall (*KRE62*, *SKO1*), morphological switching (*TPK1*, *RFX2*), biofilm (*HWP1*, *DOT5*), DNA repair and cell cycle (*RAD57*, *CSM3*, *SPC98*, *CLB4*). The genes for RT-PCR were randomly picked up from all classes and the results proved all the tested genes had good correlation with the microarray results (Fig 2B).

### PA disrupts calcineurin signaling

The transcriptome data validated by RT-PCR has revealed a link with calcineurin signaling (Fig 2). Calcineurin pathway is an effective target which acts through Ca^2+^/calmodulin activation based Ser/Thr dependent signaling pathway and responsible for virulence of *C*. *albicans* [31]. Calcineurin is a heterodimeric protein that comprises of catalytic subunit *CNA1* and regulatory subunit *CNB1*. After activation of calcineurin by Ca^2+^ it dephosphorylates and activates Crz1p, a transcription factor that encodes the products to facilitate survival of the yeast during stress conditions [32] such as alkaline pH stress, ionic stress, membrane stress, endoplasmic reticulum stress, serum stress, hyphal growth, cell wall stress [10]. Few genes associated with calcineurin signaling cascade *CNB1*, *RCN1*, *ECM7*, *VPS20*, were down regulated and *VCX1*, a gene inhibited by calcineurin was upregulated as evidenced through microarray result (Fig 2). *VCX1* is linked with tolerance and virulence through calcineurin and Ca^2+^/H^+^ exchanger in various fungi including *C*. *albicans*, *Cryptococcus neoformans*, *Aspergillus fumigates* and *Saccharomyces cerevisiae* [33–36].

**Fig 3:**
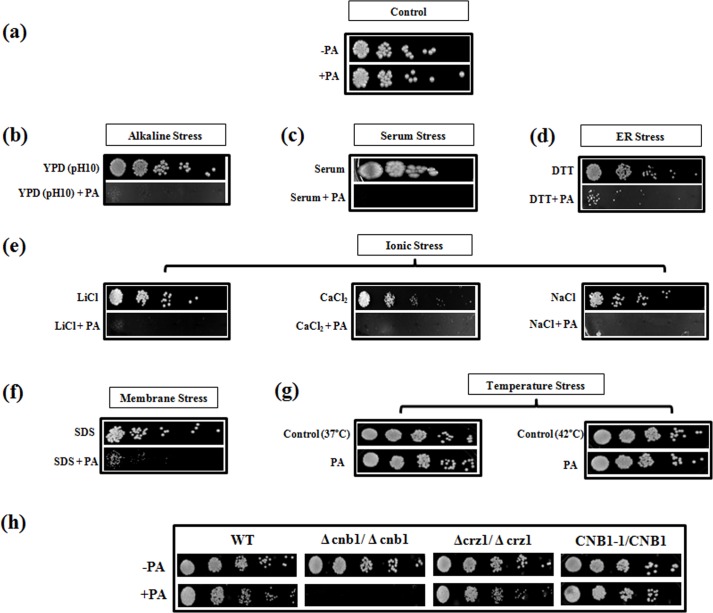
Susceptibility assays to reveal functional indispensability of calcineurin against PA in *C*. *albicans*. **(a)** Spot assay with and without PA (175 μg ml^-1^) as controls. **(b)** Spot assay with PA at alkaline pH10. **(c)** Spot assay with PA in serum (50% w/v). **(d)** Spot assay with PA under ER stress by DTT (20mM). **(e)** Spot assays with PA under various ionic stress conditions with LiCl (0.4M), CaCl_2_ (0.3M), NaCl (1M). **(f)** Spot assay with PA in presence of membrane perturbing agent SDS (0.02% w/v). **(g)** Spot assay with PA at elevated temperatures of 37°C and 42°C. **(h)** Spot assay depicting loss of growth in *Δcnb1* mutant in the presence of PA (225μg ml ^-1^) while the *Δcrz1* mutant and CNB1-1/CNB1 (calcineurin strain with constitutively expressed hyperactive allele of *CNB1*) were efficiently growing in presence of PA.

Taking clue from above observations and to verify that PA causes disruption of calcineurin pathway, we checked the response of *C*. *albicans* towards PA in the presence of different stresses that were dependent on functional calcineurin signaling cascade [18, 37]. For this, susceptibility assays were performed under serum stress, endoplasmic reticulum stress (ER stress), membrane stress, ionic stress and thermal stress. We observed that in contrast to the cells grown in absence of PA (Fig 3A), *Candida* cells with PA were hypersensitive in presence of all the calcineurin dependent stresses as depicted in Fig 3B–3F. Interestingly, at elevated temperatures (37°C and 42°C) we could not find any hypersensitive response even in presence of PA (Fig 3G). This corroborated the fact that response to thermal stress may not be governed by calcineurin in *C*. *albicans* [37].

Disruption of calcineurin signaling pathway due to PA became further apparent when calcineurin mutants such as *Δcnb1* (regulatory B subunit), *Δcrz1* and calcineurin strain having hyperactive allele (*CNB1-1/CNB1*) were tested. We observed that in contrast to the WT cells, *Δcnb1* mutant was hypersensitive to PA while *Δcrz1* mutant was not susceptible. Furthermore, the calcineurin mutant strain having constitutively expressed hyperactive allele of *CNB1* remained resistant to PA (Fig 3H). This observation corroborates our microarray and RT–PCR results which showed downregulation of *CNB1* in presence of PA (Fig 2A and 2B). Notably, transcription factor Crz1p, did not appear to have any significant role in PA mediated hypersensitivity as *Δcrz1* behaved similarly to WT cells (Fig 3H). This could be possible because Crz1p only moderately governs drug resistance downstream to calcineurin and probably has no major role in some of other effector functions [19].

**Fig 4:**
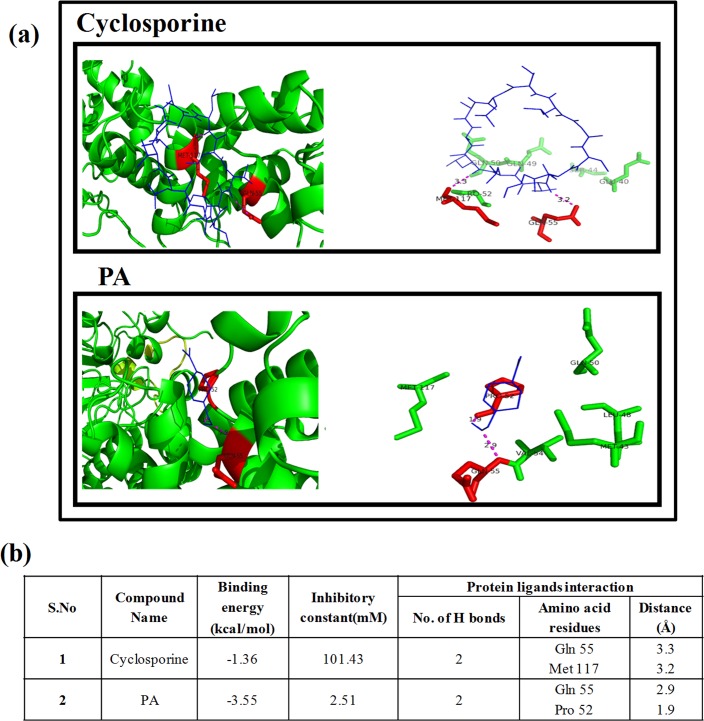
Molecular docking study on binding pattern of PA and cyclosporine (known calcineurin inhibitor) with calcineurin. **(a)** Interaction of PA (blue color ligand) binding with calcineurin (green color protein) at Gln at 55^th^ position and Pro at 52^nd^ position (depicted with red color). Interaction of cyclosporine (blue color ligand) binding with calcineurin (green color protein) at Gln at 55^th^ position and Met 117^th^ position (depicted with red color). **(b)** Binding specificities and interaction of calcineurin (Cnb1p regulatory unit) with PA.

The assumption that PA inhibits calcineurin was validated by molecular docking showing efficient binding of PA with active site of *CNB1* (Fig 4A). Our result depicts that the known calcineurin inhibitor, cyclosporine (as reference drug) had binding energy of -1.36 and inhibitory constant 101.43mM (Fig 4B). In comparison to the reference, PA had higher binding energy of -3.55 and lower inhibitory constant of 2.51mM confirming PA to be better calcineurin inhibitor. Moreover, distance between protein and ligand is lower in PA confirming an higher interaction and strong bonding of PA with the calcineurin protein. Together, all the above results clearly reveal that PA blocks calcineurin signaling pathway and functional calcineurin pathway is indispensable to sustain PA stress in *C*. *albicans*.

### Effect of PA on cell membrane

**Fig 5:**
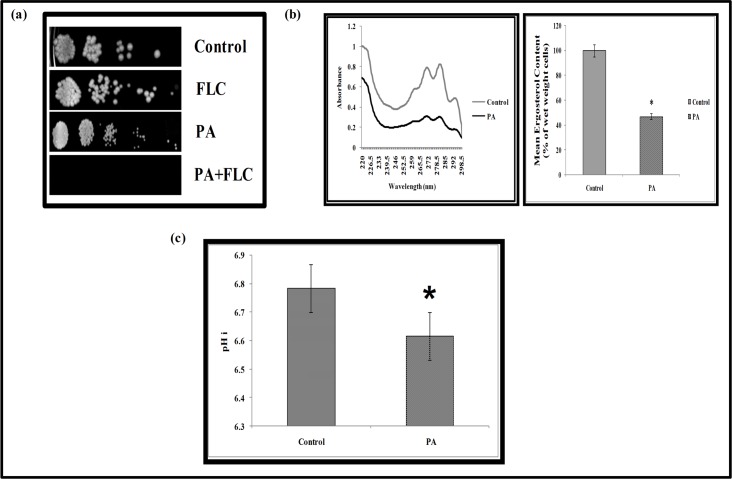
Effect of PA on cell membrane of *C*. *albicans*. **(a)** Spot assay with FLC (1μg ml^-1^), PA (175μg ml^-1^) and in combination of both. **(b)** Left panel shows UV spectrophotometric ergosterol profiles of *C*. *albicans* scanned between 220 and 300 nm from overnight culture grown in absence (control) and presence of PA (175 μg ml^-1^). Right panel shows relative percentages of ergosterol content in the absence (control) and presence of PA (175 μg ml^-1^). Mean of % ergosterol levels is calculated as described in materials and methods normalized by considering the untreated control as 100 ± SD of three independent sets of experiments is depicted on Y-axis and * depicts *P* value <0.05. **(c)** Intracellular pH (pHi) in presence of PA (MIC_80_) in *C*. *albicans* cells. Mean of pHi ± SD of three independent sets of experiments is depicted on Y-axis with respect to control & PA on X- axis and * depicts *P* value <0.05.

To decipher the role of compromised calcineurin signaling in other cellular targets they were examined closely. Several observations such as functional indispensability of calcineurin signaling to sustain PA exposure demonstrated through this study, existence of crosstalk between calcineurin and membrane stress pathways [18] and cell membrane being the most common target of mostly used antifungal drugs due to its location and functional significance, necessitated to investigate the effect of PA on cell membrane. Our microarray study revealed certain membrane function associated genes that were downregulated viz. *YKC2*, *ALG11*, *MUQ1*, *GAP6*, *ERG251*, *KAR5*, *GPB1*, *PGA33*, *SHR5*, *PGA11*, *ERG9* and *orf19*.*4982* in response to PA possibly suggesting cell membrane of *C*. *albicans* as one of the target (Fig 2A and S2 Table). This was confirmed by susceptibility assay of *Candida* cells with PA in presence of fluconazole (Fig 5A) which depicts the disruption of cell membrane by PA. The membrane perturbing effect of PA was also assessed by estimating the ergosterol levels. Interestingly, we observed a marked decrease (*P* value < 0.05) in ergosterol levels by around 53% in presence of PA (Fig 5B). This was also consistent with the fact that we found concomitant downregulation of *ERG9* and *ERG251* genes in our study (Fig 2A). Effect of PA on cell membrane was further evident by monitoring the passive diffusion of drug through cell membrane which was considerably enhanced (S2 Fig).

To further study the membrane disruption of *Candida* cells, the alteration in acidification was examined. In the present study based on microarray data, some genes such as *VMA8*, *VMA10*, *VMA13* and *VMA22* were found to be differentially regulated (Fig 2A). *VMA* class of genes are known for vacuolar ATPase activity which is responsible for several cellular processes and regulates intracellular pH in yeast by pumping cytosolic proton inside intracellular organelles [38]. Thus we ascertained whether proton pumping ability was affected which in turn may have changed internal pH (pHi). We found that only control cells with normal ATPase activity maintains the mean pHi near neutrality at 6.8 whereas PA treated cells showed decrease in mean pHi at 6.6 possibly due to increased internal acidification (Fig 5C). Thus the results clearly established that PA disrupts membrane homeostasis in *C*. *albicans*.

### Effect of PA on cell wall integrity

**Fig 6:**
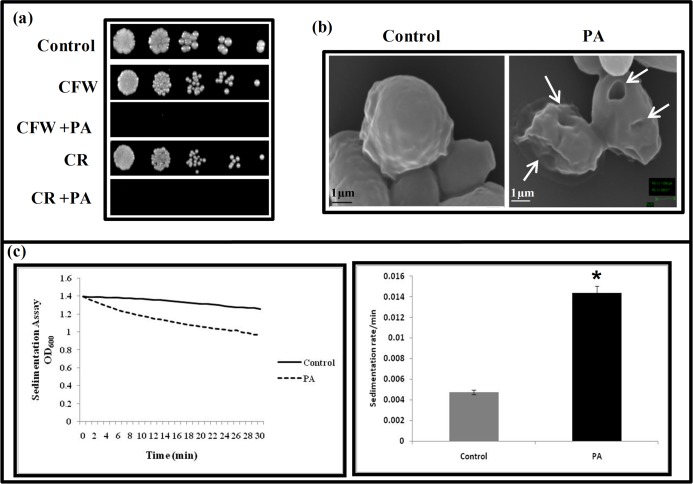
Effect of PA on the cell wall integrity of *C*. *albicans*. **(a)** Susceptibility assay showing hypersensitivity to PA (175 μg ml^-1^) in the presence of cell wall perturbing agents; CFW (10 μg ml^-1^) and CR (10 μg ml^-1^). **(b)** SEM images showing the smooth surface of untreated cell (control) and the crinkled cell wall with the leakage of its cell contents (marked with an arrow) because of the extensive damage caused due to PA. **(c)** Relative sedimentation of *C*. *albicans* cells. Left panel shows O.D_600_ of untreated (control) and PA treated (175 μg/mL) cells depicted on *y*-axis with respect to time (minutes) on *x*-axis. Right panel shows sedimentation rates per min on y-axis for control and PA on *x*-axis, calculated by estimating the difference in OD_600_ from 0 till 30 min per unit interval.

Targeting cell wall is one of the efficient ways of inhibiting the fungal growth. For instance, some of the reported natural compounds such as curcumin, chloroquine (an effective anti malarial drug), hydroxytyrosol (an antioxidant found in olive leaves and oil) target cell wall integrity (CWI) for showing antifungal activity [39–41]. CWI pathway is known to regulate key cellular responses crucial for survival from exposure to antifungal drugs targeting the cell wall and also linked with calcineurin signaling suggesting crosstalk [39, 42, 43]. Our microarray data revealed prominent changes in the genes linked to CWI such as *KRE62*, *PGA25*, *CWH41*, *HWP1*, *SKO1*, *MUM3* that were down regulated (Fig 2A). To confirm the effect of PA on CWI, we performed susceptibility assays in presence of cell wall damaging agents viz. CFW and CR. We observed *C*. *albicans* cells were unable to grow in presence of PA when administered with either CFW or CR (Fig 6A). The cell wall damaging effect was also confirmed by visualizing the PA treated *Candida* cells with SEM which revealed wrinkling and corrugation of cell surface morphology as compared with the untreated cells (Fig 6B). Furthermore, for a comparative analysis of cell wall damage, we used a spectrophotometric assay to measure cell sedimentation rates. Interestingly, sedimentation rate of cells grown in the presence of PA was almost three times faster in comparison to the control (Fig 6C) supporting the notion that cell clustering is affected during the membrane and cell wall stress. Moreover, this observation was also consistent with the downregulation of *CHT3* observed in our study (Fig 2A). The cell wall perturbation and downregulation of *CHT3* gene is known to be directly linked with the increased sedimentation rate of the cells [24]. These facts confirm that the anticandidal effects of PA are also associated with disruption of CWI. However, whether cell wall damaging effect of PA is due to compromised calcineurin pathway or independent mechanism requires further validation.

### Effect of PA on hyphal morphogenesis

**Fig 7:**
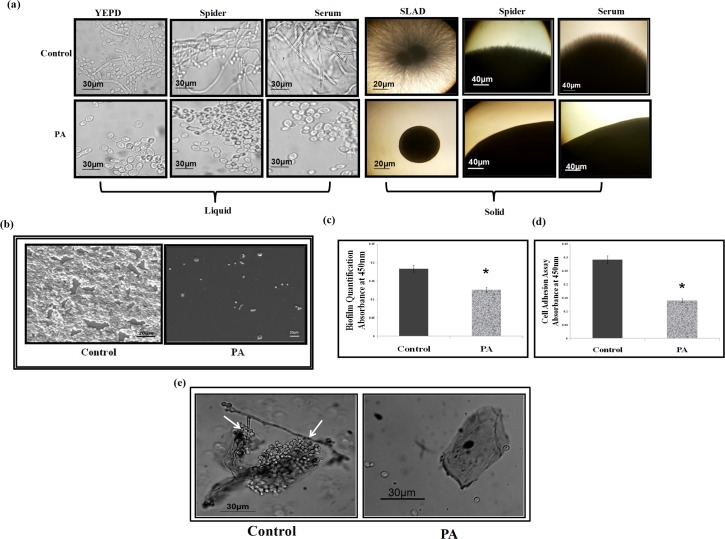
Effect of PA on virulence traits of *C*. *albicans*. **(a)** Effect of PA on Dimorphic Switching of *C*. *albicans*. Left panel shows hyphal morphogenesis in the liquid hyphal inducing media (YEPD, Spider media and YEPD with 10% Serum) in the absence (control) and presence of PA (225μg ml^-1^) in *C*. *albicans* at 4 hours (Magnification 40X). Right panel shows hyphal morphogenesis in the solid hyphal inducing media (SLAD medium, Spider medium and 10% Serum) in the absence (control) and presence of PA (225μg ml^-1^) in *C*. *albicans* at 4 hours (Magnification 4X). **(b)** SEM showing the biofilm formation in absence (control) and presence of PA (175μg ml^-1^). **(c)** Effect of PA (175μg ml^-1^) on biofilm formation of *C*. *albicans* depicted as bar graph and quantified by using MTT assay as described in material and methods. Mean of OD_450_ ± SD of three independent sets of experiments is depicted on Y-axis and * depicts *P* value <0.05. **(d)** Effect of PA (175μg ml^-1^) on cell adherence of *C*. *albicans* quantified by using MTT assay as described in material and methods. Mean of O.D_450_ ± SD of three independent sets of experiments is depicted on Y-axis and * depicts *P* value <0.05. **(e)** Effect of PA (175μg ml^-1^) on adherence of *C*. *albicans* to the buccal mucosal epithelial cell. Left panel shows *Candida* cells adhered to the epithelial cells marked by an arrow and inhibitory effect of PA showing no adherence in right panel.

Yeast to hyphal switching in *C*. *albicans* is one of the major factors governing the virulence and this transition is known to be directly linked to the calcineurin pathway in various pathogenic fungi [37]. Our microarray data revealed that some genes responsible for the morphogenetic switching of *C*. *albicans* were differentially affected viz, *GCN4*, *TPK1*, *RFX2*, *VPS41*, *FGR6-4*, *IHD2*, *TCC1*, *UEC1*, *IFE2*, *SPT6*, *RNY11* (Fig 2A). Therefore, we scrutinized the effect of PA on this morphological switching in presence of various hyphae inducing conditions at 37°C. We found that PA treated cells completely lacked filamentation and appeared only in yeast form in contrast to the untreated *Candida* cells which were efficiently able to express hyphal form suggesting that PA is an active inhibitor of the morphogenetic switching (Fig 7A). The role of calcineurin in yeast to hyphal transition of *C*. *dubliniensis* and *C*. *lusitaniae* is well established [44, 45]. However, it is still unclear in *C*. *albicans*, as there is evidence which endorses the role of calcineurin for hyphal formation in *C*. *albicans* [46] while there exist reports which suggest otherwise [47, 48]. From our study, role of compromised calcineurin signaling due to PA being the main reason for inhibited morphogenetic switching cannot be ruled out.

### Effect of PA on biofilm formation and cell adhesion

A striking and clinically relevant virulence trait of *C*. *albicans* is its ability to form biofilms. The inhibited yeast to hyphal transition in response to PA observed in this study and functional morphogenetic switching as a prerequisite for biofilm formation [49, 50, 51] made us to try elucidate the effect of PA on biofilm formation of *C*. *albicans*. Moreover, the microarray data revealed down regulation in several important genes that were linked to the biofilm formation and cell adhesion such as *DOT5*, *PUT1*, *PUT2*, *HSP104*, *CFL2*, *YCP4* and *HWP1*(Fig 2A). Biofilm formation was firstly visualized by performing SEM in the absence (control) and presence of PA which depicts disruption in biofilm formation (Fig 7B). This result was further validated quantitatively by performing MTT assay which showed that biofilm formation was considerably (*P* < 0.05) inhibited by more than 30% in presence of PA in *C*. *albicans* (Fig 7C). Cell adherence is a primary step in formation of biofilm where *C*. *albicans* adhere to the substratum and extended extracellular matrix is assembled in the maturation step to develop the biofilm [50, 51]. So we tried to verify whether lack of biofilm formation in presence of PA is due to the inhibition of cell adherence. Cell adherence was observed to be significantly (*P* < 0.05) decreased on polystyrene surface by 52% in presence of PA (Fig 7D) showing it to be an efficient inhibitor of cell adherence in *C*. *albicans*.

### Effect of PA on adherence of *C*. *albicans* to buccal epithelial cells

To further substantiate the effect of PA on the adherence of *C*. *albicans*, an assay was performed using buccal epithelial cells [25]. Fig 7E clearly depicts that in contrast to the control where *Candida* cells adhered to the epithelial cells and only a few cells were left in the surrounding, PA treated *Candida* cells showed almost no adherence to the epithelial cells reinforcing our observation that PA leads to loss of adherence. By virtue of the ability of PA to block the virulence traits in *C*. *albicans*, further investigations are warranted.

### Effect of PA on DNA repair and cell cycle

**Fig 8:**
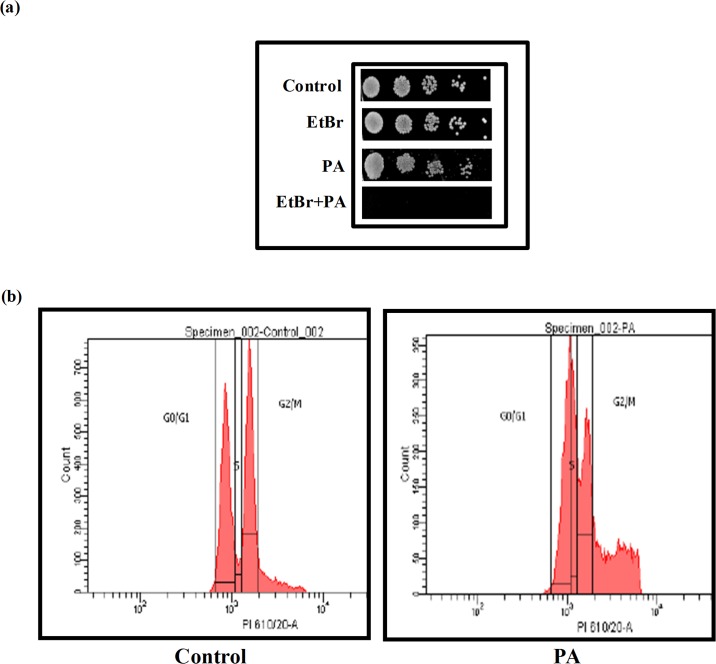
Effect of PA on DNA repair and cell cycle. (a) Spot assay in absence (control) and presence of PA (175μg ml^-1^) depicting loss of growth with DNA damaging agent EtBr (50μg ml^-1^). (b) Cell cycle analysis of control and PA (175μg ml^-1^) treated *C*. *albicans* by Flow Cytometry.

Our microarray data revealed that several genes associated with DNA repair and cell cycle inhibition in *C*. *albicans* were differentially affected in response to PA *viz*. *MSH6*, *CSM3*, *RAD57*, *TFB1*, *NUP120*, *DNA2*, *RAD10*, *RAD52*, *ESC2*, *RAD3*, *SPC98*, *CLB4*, *TVP38*, *BIR1*, *CDC16*, *DLH1*, *RRD2*, *RSC8*, *BZZ1*, *STS1*, *HAT2*, *YMR262W*, *POL12*, *VIP1*, *YIH1*, *TRA1*, *CAP2*, *TUB4*, *SET5*, *MSC7*, *MCD1*, *SLD2*, *ORC5*, *PCL7* (Fig 2A). Therefore, effect of PA on DNA repair of *C*. *albicans* was evaluated. For this, *Candida* cells were spotted with PA in presence of EtBr, a known DNA damaging agent, at a concentration that showed no appreciable growth defect. The result depicts that *C*. *albicans* was unable to grow in presence of PA when spotted in combination with EtBr suggesting the inhibitory effect of PA may be related to some defect in DNA repair mechanisms in *C*. *albicans* (Fig 8A). This result was further confirmed by FACS analysis which showed that the number of cells arrested in S phase was 12.5% more in PA treated cells than the control (drug free) cells while the number of cells in G_0_/G_1_ and G_2_/M phase were decreased in PA treated cells in comparison to control by 14.5% and 15.6% respectively (Fig 8B). SWE1p, is a Wee1-family kinase that is expressed during late G1 and S phase and prevents the cell to enter both mitotic and the isotropic shift [52]. Since, Swe1p stabilization endorses cell cycle arrest, its upregulation as revealed in our study is only be fitting (Fig 2A). Similarly, *RAD52* is the gene known to play role in the homologous recombination, genomic stability and DNA repair in *C*. *albicans* [53, 54], was downregulated in our study (Fig 2A). *CLB4*, is a homolog of *CLB5/CLB6* which is S-phase specific cyclin in *S*. *cerevisiae* [55], was also downregulated in PA treated cells supporting our FACS data which depicts cell cycle arrest at S phase (Fig 8B). Thus downregulation of genes associated with DNA repair and cell cycle, inability to grow in presence of DNA damaging agent and the cell cycle arrest of *C*. *albicans* confirms that PA obstructs the DNA repair machinery and cell cycle in *C*. *albicans*.

### Effect of PA on mitochondrial activity

**Fig 9:**
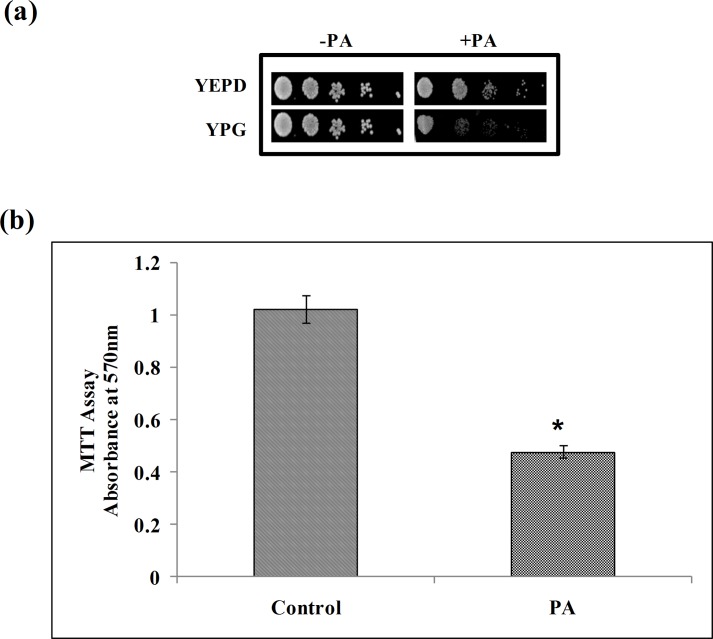
Effect of PA on mitochondria activity. **(a)** Spot assays in absence and presence of PA (175 μg ml^-1^) in YEPD (fermentable carbon source) and YPG media (non-fermentable carbon source). **(b)** Effect of PA (175μg ml^-1^) on mitochondrial activity of *C*. *albicans* depicted as bar graph and quantified by using MTT assay as described in material and methods. Mean of O.D_570_ nm ± SD of three independent sets of experiments is depicted on Y-axis and * depicts P value <0.05.

Our microarray data clearly revealed various mitochondrial genes such as *ILM1*, *COX6*, *OAR1*, *PIM1*, *ISD11*, *GDH2*, *FMP30*, *MRP21*, *SEN54*, *VMS1*, *SLM3*, *MAM33*, *AIM25*, *EHT1*, *NAM2*, *FCJ1*, *PET54*, *MTG1*, *PIM1*, *KTI11*, *MRPL44*, *MAE1*, *CYB2*, *TOM1* that were differentially regulated in response to PA (Fig 2A). Hence we examined whether or not the anticandidal effect of PA is linked with compromised mitochondrial activity. This was confirmed by spotting *C*. *albicans* on YPG medium, having non-fermentable carbon source, in presence of PA. Hypersensitivity of *C*. *albicans* on YPG medium in the presence of PA, in contrast to control cells which were growing efficiently without PA in the same medium, confirmed the dysfunction of mitochondria (Fig 9A). To further validate our observations, we quantitatively analyzed the mitochondrial activity through MTT assay, a commonly used indicator of cellular metabolic activity, which is reduced to colored formazan by mitochondrial enzymes and electron carriers correlating with mitochondrial activity [28]. To our expectation, we found the mitochondrial activity was considerably reduced to 53% in the presence of PA (Fig 9B) reinforcing that PA abrogates the mitochondrial activity in *C*. *albicans*. Considering the emerging role of mitochondria in MDR of *C*. *albicans* [56] further work is needed to reveal the precise mechanisms by which PA causes mitochondrial dysfunction.

## Conclusion

**Fig 10:**
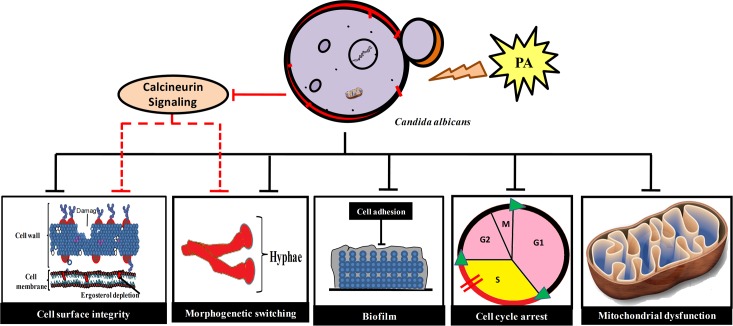
Summary of PA mode of action in *C*. *albicans*. PA blocks calcineurin signaling, affects cell surface integrity (cell wall and cell membrane), yeast to hyphal transition, biofilm formation, cell cycle arrest at S phase and mitochondria dysfunction (shown by dark lines). Abrogated membrane homeostasis, cell wall integrity, yeast to hyphal transition could also be the result of compromised calcineurin signaling because of PA (denoted by red dashed lines). All these disrupted mechanisms due to PA are necessary to sustain drug tolerance and virulence in *C*. *albicans*.

Natural compounds like PA that have potential to be used as antifungal agents have become a rehabilitated source of interest in the era of ever increasing MDR burden. Our data has showed enough promising antifungal potential of PA with its multiple mechanisms of actions (Fig 10) to draw attention for further investigations. May be further chemical modification could be an attractive approach to increase its potency which could further be helpful in lowering the dosages of present antifungals in combination. Together, natural chemosensitizing agent such as PA presents an opportunity to employ this new information in improving the antifungal treatment strategies.

## Supporting Information

S1 Fig Growth Curve of *C*. *albicans* in response to PA. The growth of the cells depicted by measuring absorbance at 600nm (γ-axis) with respect to time in hours (*x*-axis) in the absence (control) and presence of 175, 225 and 350μg/ml PA.
(DOC)

S2 Fig Drug efflux assay and passive diffusion in presence of PA. Extracellular concentrations of R6G for *C*. *albicans* (SC5314) cells grown in absence (control) and presence of PA (175 μg ml^-1^). For passive diffusion and efflux assay the *C*. *albicans* (SC5314) cells cultured overnight at 30°C in absence (control) and presence of PA (175 μg ml^-1^) were harvested, washed and resuspended in 2% cell suspension with PBS containing 5mM 2-DOG and 5mM 2,4 DNP for 1h to de-energize the cells and subsequently harvested at 5000xg for 3 min. The harvested cells were washed and resuspended in PBS 2% (w/v) with 10μM R6G for 40 min. After washing with PBS (-Glu) the cells were centrifuged at 10,000xg for 1 min and OD_527_ of the supernatant were measured at indicated time points for passive diffusion of R6G. For efflux assay, the penultimate step washing is done with PBS (+Glu) after 10 min (indicated by arrow) and then the cells were centrifuged at 10,000xg for 1 min and OD_527_ of the supernatant were measured at indicated time points. Mean of OD_527_ ± SD of three independent sets of experiments are depicted on γ-axis with respect to time (minutes) on *x*-axis.
(DOC)

S1 Table List of primers used for RT-PCR in the study. (DOCX)

S2 Table List of downregulated genes in response to PA. (DOCX)

S3 Table List of upregulated genes in response to PA. (DOCX)

## Abbreviations

PA: Perillyl alcohol
R6G: Rhodamine 6G
2-DOG: 2-Deoxy D-glucose
2,4 DNP: 2,4 Dinitrophenol
CFW: Calcoflour white
CR: Congo Red
YEPD: Yeast Extract Peptone Dextrose
SEM: Scanning Electron Microscopy
