# Supplementary figures and images for: Anticandidal Effect and Mechanisms of Monoterpenoid, Perillyl Alcohol against Candida albicans
Source: PLoS One. 2016 Sep 14;11(9):e0162465. doi: 10.1371/journal.pone.0162465 (PMC5023166; doi:10.1371/journal.pone.0162465)

**S1 Fig.**

**
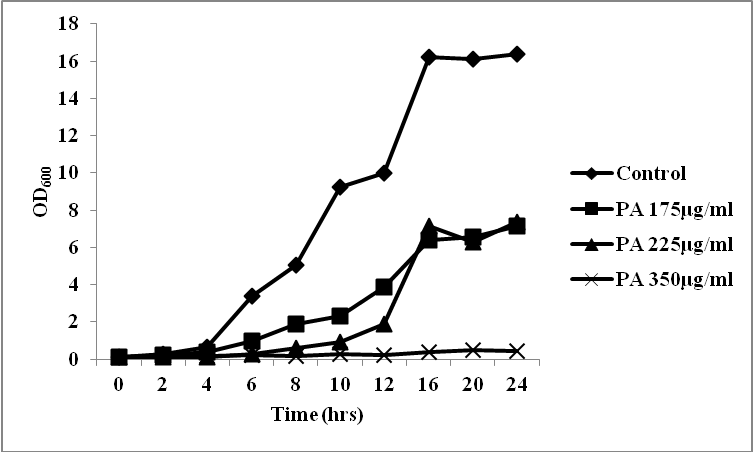
**

Supplement: S1 Fig — The growth of the cells depicted by measuring absorbance at 600nm (γ-axis) with respect to time in hours (x-axis) in the absence (control) and presence of 175, 225 and 350μg/ml PA. (DOC) [file pone.0162465.s001.doc]

**S2 Fig.**

**
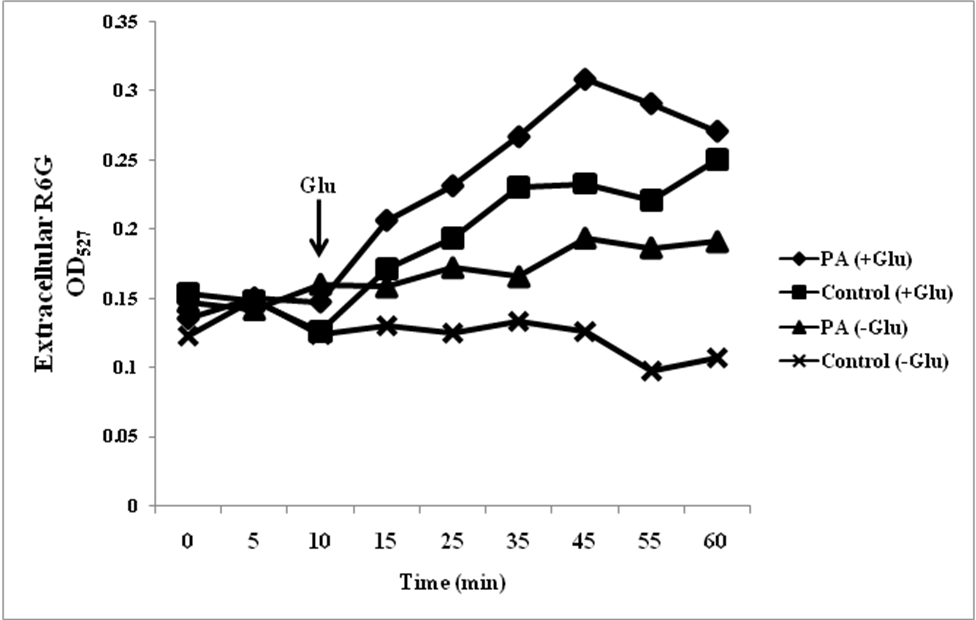
**

Supplement: S2 Fig — Extracellular concentrations of R6G for C. albicans (SC5314) cells grown in absence (control) and presence of PA (175 μg ml-1). For passive diffusion and efflux assay the C. albicans (SC5314) cells cultured overnight at 30°C in absence (control) and presence of PA (175 μg ml-1) were harvested, washed and resuspended in 2% cell suspension with PBS containing 5mM 2-DOG and 5mM 2,4 DNP for 1h to de-energize the cells and subsequently harvested at 5000xg for 3 min. The harvested cells were washed and resuspended in PBS 2% (w/v) with 10μM R6G for 40 min. After washing with PBS (-Glu) the cells were centrifuged at 10,000xg for 1 min and OD527 of the supernatant were measured at indicated time points for passive diffusion of R6G. For efflux assay, the penultimate step washing is done with PBS (+Glu) after 10 min (indicated by arrow) and then the cells were centrifuged at 10,000xg for 1 min and OD527 of the supernatant were measured at indicated time points. Mean of OD527 ± SD of three independent sets of experiments are depicted on γ-axis with respect to time (minutes) on x-axis. (DOC) [file pone.0162465.s002.doc]
