# Supplementary material for: Anticandidal Effect and Mechanisms of Monoterpenoid, Perillyl Alcohol against Candida albicans
Source: PLoS One. 2016 Sep 14;11(9):e0162465. doi: 10.1371/journal.pone.0162465 (PMC5023166; doi:10.1371/journal.pone.0162465)
Supplement: S1 Table — (DOCX) [file pone.0162465.s003.docx]

**S1 Table. List of primers used for RT–PCR in the study.**

| **Sr. No.** | **Gene name** | **Primer Sequence** |
| --- | --- | --- |
|  | ACT1 | **F:**TTTTGACCTTGAGATACCCA  **R:**GGAGCTCTGAATCTTTCGTT |
|  | CNB1 | **F:**ATGGGGGCTAATGCAAGTAT  **R:**AATGTCAAAGTGTTGGCAAT |
|  | VCX1 | **F:**TTGGGTATTATTGCTGGG  **R:**CAAGTAAACGATCAACAAGA |
|  | NPC2 | **F:**GAACTTGGCAATTGTTACCC  **R:**CAGGGAATATAATTGTAGCAG |
|  | KRE62 | **F:**AAGAAGTCTGGTGGGAAA  **R:**CCATAATAAAGTTCCACCGC |
|  | SKO1 | **F:**AATTACCAGGTATTACGCCAC  **R:**ACTCAGAGGGGTTTGTGTTA |
|  | GLN3 | **F:**TAGGTGCCACACATGATAAA  **R:**TATGGCTATTGGCGTTTG |
|  | TPK1 | **F:**ATGGAACCAGCAGACACAA  **R**:CCCCCACATTCGAATTAT |
|  | RFX2 | **F:**AACAACATCTACCACACATCC  **R:**CTGATGTTGTCGTAAAGCAG |
|  | HWP1 | **F:**ACTACCCACAACAACCACAA  **R:**GCAGATGATGATTCTGAAGTG |
|  | DOT5 | \| **F:**ACAACCACCTACCAAGAAAGT \| \| --- \| \| **R:**GGCACTTGTAAAGCTGACTT \| |
|  | RAD57 | **F:**AAAAGGATTTGCAAGTCG  **R:**GAATCTTCTCTCCGGAAA |
|  | CSM3 | **F:**ATAGAGACTCCTCGACCCAA  **R:**GGCTGCTATTAATTCTCTTC |
|  | SPC98 | **F:**CTTTATCGGAACAAACCA  **R:**CCCTTTCACTATCCAATGTT |
|  | CLB4 | **F:**CAGTGCTTATTGATTGGGTC  **R:**TCTTCCCAATAGTATTCTTGA |
