# Supplementary material for: Anticandidal Effect and Mechanisms of Monoterpenoid, Perillyl Alcohol against Candida albicans
Source: PLoS One. 2016 Sep 14;11(9):e0162465. doi: 10.1371/journal.pone.0162465 (PMC5023166; doi:10.1371/journal.pone.0162465)
Supplement: S2 Table — (DOCX) [file pone.0162465.s004.docx]

**S2 Table. List of downregulated genes in response to PA.**

| **ORF ID^¥^** | **Standard**  **gene name^¥^** | | **Fold change^*^(Log_2_)** | | **Description** |
| --- | --- | --- | --- | --- | --- |
| **Plasma membrane genes** | | | | | |
| orf19.7001 | YCK2 | | -3.7 | | Plasma membrane protein similar to *S. cerevisiae*  casein kinase I, Yck2p; null mutant has defect in damaging oral epithelial cells and in hyphal branching; transcription is activated in weak acid stress or on contact with host cells |
| orf19.3468 | ALG11 | | -1.6 | | Ortholog(s) have alpha-1,2-mannosyltransferase activity, role in oligosaccharide-lipid intermediate biosynthetic process and endoplasmic reticulum membrane localization |
| orf19.4982 |  | | -3.2 | | Has domain(s) with predicted role in lipid metabolic process |
| orf19.2107 | MUQ1 | | -2.1 | | Putative choline phosphate cytidylyltransferase / phosphoethanolamine-cytidylyltransferase; decreased expression in hyphae compared to yeast-form cells; Hap43p-repressed gene; biofilm-induced |
| orf19.6659 | GAP6 | | -4.1 | | Broad-specificity amino acid permease; Plc1p-regulated; Gcn4p-regulated; fungal-specific (no human or murine homolog) |
| orf19.4631 | ERG251 | | -1.5 | | C-4 sterol methyl oxidase with a role in ergosterol biosynthesis; Hap43p-induced; ketoconazole-induced; amphotericin B, caspofungin repressed; possibly an essential gene, disruptants not obtained by UAU1 method |
| orf19.103 | KAR5 | | -2.4 | | Protein similar to *S. cerevisiae*  Kar5p, involved in nuclear membrane fusion during karyogamy; induced by alpha factor |
| orf19.4577 | GPB1 | | -1.5 | | Ortholog(s) have GTPase activating protein binding, cAMP-dependent protein kinase inhibitor activity, signal transducer activity |
| orf19.876 | PGA33 | | -1.5 | | Putative GPI-anchored protein |
| orf19.1955 | SHR5 | | -2.1 | | Ortholog(s) have palmitoyltransferase activity, role in protein palmitoylation, protein targeting to membrane and endoplasmic reticulum palmitoyltransferase complex, extrinsic to endoplasmic reticulum membrane localization |
| orf19.7609 | PGA11 | | -2.3 | | Putative GPI-anchored protein |
| orf19.3616 | ERG9 | | -1.6 | | Putative farnesyl-diphosphate farnesyl transferase (squalene synthase) involved in the sterol biosynthesis pathway; likely to be essential for growth; regulated by fluconazole and lovastatin; amphotericin B, caspofungin repressed |
| **Yeast to Hypha transition** | | | | | |
| orf19.1358 | GCN4 | | -1.3 | | Transcriptional activator of general amino acid control response; required for Efg1p-dependent pseudohyphal induction by amino acid starvation, not by serum; required for normal biofilm formation; upregulated by human whole blood and PMN |
| orf19.4892 | TPK1 | | -4.0 | | Catalytic subunit of cAMP-dependent protein kinase (PKA), isoform of Tpk2p; involved in control of morphogenesis and stress response; wild-type nuclear localization requires Bcy1p; produced during stationary but not exponential growth |
| orf19.4590 | RFX2 | | -1.9 | | Transcriptional repressor; regulator of filamentation, response to DNA damage, adhesion, virulence in murine mucosal, systemic infections; RFX domain; regulated by Nrg1p, UV-induced; partially complements *S. cerevisiae*  rfx1 mutant defects |
| orf19.4858 | VPS41 | | -1.8 | | Protein similar to *S. cerevisiae*  Vps41p, which is involved in vacuole organization and biogenesis; transposon mutation affects filamentous growth; regulated by Gcn4p; induced in response to amino acid starvation (3-aminotriazole treatment) |
| orf19.3490 | FGR6-4 | | -2.2 | | Protein lacking an ortholog in *S. cerevisiae* ; member of a family encoded by FGR6-related genes in the RB2 repeat sequence; transposon mutation affects filamentous growth |
| orf19.6021 | IHD2 | | -1.6 | | Transcriptionally regulated by iron and by yeast-hyphal switching; expression greater in low iron |
| orf19.6734 | TCC1 | | -1.6 | | Protein involved in regulation of filamentous growth and virulence; interacts with Tup1p; acts in transcriptional regulation of hypha-specific genes; contains 4 tetratricopeptide repeat (TPR) motifs; flucytosine repressed; Tbp1p-activated |
| orf19.4646 | UEC1 | | -1.7 | | Protein required for damage to oral epithelial cells and for normal hyphal growth and stress resistance; transcription induced on contact with vascular endothelial cells; not highly conserved |
| orf19.5288 | IFE2 | | -2.0 | | Putative alcohol dehydrogenase; decreased expression in hyphae compared to yeast-form cells; Efg1p-regulated; fluconazole-induced; Hog1p-induced; increased expression in response to prostaglandins |
| orf19.7136 | SPT6 | | -2.3 | | Protein similar to *S. cerevisiae*  Spt6p transcription elongation factor; transposon mutation affects filamentous growth; transcription is upregulated in an RHE model of oral candidiasis and in clinical isolates from oral candidiasis |
| orf19.3926 | RNY11 | | -1.6 | | Ortholog(s) have endoribonuclease activity, role in RNA catabolic process, apoptotic process, cell morphogenesis and cytosol, extracellular region, vacuole localization |
| **Biofilm formation** | | | | | |
| orf19.4274 | PUT1 | | -1.9 | | Putative proline oxidase; alkaline upregulated by Rim101p; biofilm-induced |
| orf19.1321 | HWP1 | | -1.8 | | Hyphal cell wall protein; covalently crosslinked to epithelial cells by host transglutaminase; opaque- and a-specific, alpha-factor induced; at MTLa side of conjugation tube; assessment of virulence role complicated by URA3 effects |
| orf19.6387 | HSP104 | | -2.7 | | Heat-shock protein; functional homolog of *S. cerevisiae*  Hsp104p; chaperone and prion propagation activity in *S. cerevisiae* ; guanidine-insensitive; heat shock/stress induced; downregulated in farnesol-treated biofilm; sumoylation target |
| orf19.1264 | CFL2 | | -2.4 | | Putative oxidoreductase, iron utilization; regulated by Sfu1p, Sef1p, Hap43p, Nrg1p, Tup1p, Rim101p; alkaline, low iron, fluphenazine, ciclopiroxolamine, flucytosine, fluconazole, biofilm induced; caspofungin, amphotericin B repressed |
| orf19.5417 | DOT5 | | -2.98 | | Putative nuclear thiol peroxidase; alkaline downregulated; biofilm- and planktonic growth-induced gene; sumoylation target |
| orf19.3974 | PUT2 | | -1.5 | | Putative delta-1-pyrroline-5-carboxylate dehydrogenase; alkaline upregulated; protein present in exponential and stationary growth phase yeast cultures; late-stage biofilm-induced gene |
| orf19.5286 | YCP4 | | -2.1 | | Putative flavodoxin; fungal-specific (no human or murine homolog) |
| **Calcineurin signaling** | | | | | |
| orf19.4009 | CNB1**^¶^** | | -1.4 | | Regulatory subunit of calcineurin B (Ca[2+]-calmodulin-regulated S/T protein phosphatase); required for wild-type resistance to fluconazole or to SDS; micafungin is fungicidal to null mutant |
| orf19.123 | RCN1 | | -1.6 | | Protein involved in calcineurin-dependent signaling that controls stress response and virulence; inhibits calcineurin function |
| orf19.5643 | ECM7 | | -2.0 | | Ortholog(s) have role in calcium ion transport, cellular response to drug, fungal-type cell wall organization |
| orf19.897 | VPS20 | | -1.9 | | ESCRT III complex protein with a role in multivesicular body (MVB) trafficking; required for processing of Rim8p |
| **Cell wall** | | | | | |
| orf19.942 | KRE62 | | -2.2 | | Putative subunit of glucan synthase; macrophage-induced gene |
| orf19.6336 | PGA25 | | -1.9 | | Putative GPI-anchored adhesin-like protein; fluconazole-downregulated; induced in oralpharyngeal*Candida*sis |
| orf19.4719 | CWH41 | | -1.5 | | Processing alpha glucosidase I, involved in N-linked protein glycosylation and assembly of cell wall beta 1,6 glucan |
| orf19.1032 | SKO1 | | -1.5 | | bZIP domain transcription factor involved in cell wall damage response; represses the yeast-to-hypha transition; mutants are caspofungin sensitive; induced by osmotic stress via Hog1p; induced by Mnl1p under weak acid stress |
| orf19.1647 | MUM3 | | -1.7 | | Ortholog(s) have role in ascospore wall assembly |
| **Iron** | | | | | |
| orf19.2198 | FLC3 | | -1.5 | | Protein involved in heme uptake; putative FAD transporter, similar to *S. cerevisiae*  Flc3p |
| **Cell cycle arrest/Cytoskeleton organisation/DNA replication/DNA repair** | | | | | |
| orf19.2600 | SPC98 | | -3.4 | | Putative component of the microtubule-nucleating Tub4p (gamma-tubulin) complex; periodic mRNA expression, peak at cell-cycle S/G2 phase |
| orf19.5534 | TVP38 | | -2.6 | | Ortholog(s) have role in mitotic spindle elongation, vesicle-mediated transport and integral to Golgi membrane localization |
| orf19.643 | BIR1 | | -2.7 | | Ortholog(s) have role in apoptotic process, mitotic spindle elongation, sister chromatid biorientation, spindle checkpoint |
| orf19.1792 | CDC16 | | -2.3 | | Ortholog(s) have ubiquitin-protein ligase activity |
| orf19.3760 | DLH1 | | -3.2 | | Functional homolog of *S. cerevisiae*  Dmc1p, which is a meiosis-specific protein |
| orf19.6933 | RRD2 | | -2.7 | | Ortholog(s) have peptidyl-prolyl cis-trans isomerase activity, protein phosphatase type 2A regulator activity and role in mitotic spindle organization in nucleus, response to osmotic stress |
| orf19.7234 | RSC8 | | -2.5 | | Putative RSC chromatin remodeling complex component; possibly an essential gene, disruptants not obtained by UAU1 method |
| orf19.1699 | BZZ1 | | -3.4 | | Protein similar to *S. cerevisiae*  Bzz1p, which is an SH3 domain protein involved in the regulation of actin polymerization |
| orf19.4849 | STS1 | | -1.6 | | Planktonic growth-induced gene; possibly an essential gene, disruptants not obtained by UAU1 method |
| orf19.2146 | HAT2 | | -1.5 | | Ortholog(s) have histone acetyltransferase activity, histone binding activity, role in chromatin assembly or disassembly, chromatin silencing at telomere, histone acetylation and cytoplasm, histone acetyltransferase complex localization |
| orf19.6744 | YMR262W | | -1.7 | | Ortholog(s) have role in regulation of mitotic metaphase/anaphase transition and cytosol, nucleus localization |
| orf19.2796 | POL12 | | -1.8 | | Ortholog(s) have DNA-directed DNA polymerase activity, role in DNA-dependent DNA replication initiation, telomere capping and alpha DNA polymerase:primase complex, cytosol, nuclear envelope localization |
| orf19.980 | VIP1 | | -2.5 | | Ortholog(s) have inositol heptakisphosphate kinase activity, inositol hexakisphosphate 1-kinase activity, inositol hexakisphosphate 3-kinase activity, inositol hexakisphosphate 4-kinase activity, inositol hexakisphosphate 6-kinase activity |
| orf19.3273 | YIH1 | | -1.8 | | Ortholog(s) have actin monomer binding, protein kinase inhibitor activity, ribosome binding activity, role in negative regulation of protein phosphorylation and cytoplasm, nucleus, polysome, ribosome localization |
| orf19.7186 | CLB4 | | -1.7 | | B-type mitotic cyclin; nonessential; negative regulator of pseudohyphal growth; dispensible for mitotic exit, cytokinesis; increased CLB4 transcript detected in fkh2 mutant; farnesol-upregulated in biofilm; reduced total RNA in clb4 mutant |
| orf19.139 | TRA1 | | -1.4 | | Subunit of the NuA4 histone acetyltransferase complex |
| orf19.4597 | CAP2 | | -1.8 | | Putative F-actin-capping protein subunit beta; possibly an essential gene, disruptants not obtained by UAU1 method |
| orf19.1238 | TUB4 | | -1.6 | | Putative gamma-tubulin; induced upon adherence to polystyrene; transcription is regulated by Nrg1p and Mig1p; periodic mRNA expression, peak at cell-cycle S/G2 phase |
| orf19.1972 | SET5 | | -2.9 | | Ortholog(s) have S-adenosylmethionine-dependent methyltransferase activity, role in histone lysine methylation and cytoplasm, nuclear chromatin localization |
| orf19.1865 | MSC7 | | -1.8 | | Ortholog(s) have role in reciprocal meiotic recombination and cytosol, endoplasmic reticulum, nucleus localization |
| orf19.7634 | MCD1 | | -1.7 | | Protein similar to *S. cerevisiae*  Mcd1p; transcription is repressed in response to alpha pheromone in SpiderM medium; cell-cycle regulated periodic mRNA expression; Hap43p-induced |
| orf19.2389 | SLD2 | | -2.0 | | Ortholog(s) have DNA replication origin binding, DNA strand annealing activity |
| orf19.2369 | ORC5 | | -2.0 | | Ortholog(s) have ATP binding, DNA replication origin binding activity |
| orf19.6225 | PCL7 | | -1.6 | | Putative cyclin-like protein, possible Pho85p cyclin; hyphal downregulated; transcriptionally activated by Mnl1p under weak acid stress |
| orf19.4945 | MSH6 | | -3.2 | | Protein similar to *S. cerevisiae*  Msh6p, which is involved in mismatch repair; repressed under Cdc5p depletion; Hap43p-induced gene |
| orf19.4105 | CSM3 | | -4.2 | | Ortholog(s) have role in DNA repair, DNA replication checkpoint, establishment of mitotic sister chromatid cohesion, maintenance of DNA repeat elements, meiotic chromosome segregation, replication fork arrest, replication fork protection |
| orf19.2174 | RAD57 | | -2.0 | | Putative DNA recombination and repair protein; transcriptionally induced by interaction with macrophage; transcription is regulated by Nrg1p, Mig1p, and Tup1p; essential protein; *S. cerevisiae*  ortholog is essential |
| orf19.5297 | TFB1 | | -2.2 | | Ortholog(s) have phosphatidylinositol-3-phosphate binding, phosphatidylinositol-5-phosphate binding activity |
| orf19.4627 | NUP120 | | -1.6 | | Ortholog(s) have structural constituent of nuclear pore activity |
| orf19.1192 | DNA2 | | -1.8 | | Protein similar to *S. cerevisiae*  Dna2p, which is a DNA replication factor involved in DNA repair; induced under hydroxyurea treatment |
| orf19.2469 | RAD10 | | -1.6 | | Ortholog of *S. cerevisiae*  RAD10, an endonuclease involved in nucleotide excision repair; null mutant is extremely sensitive to UV irradiation; transcription is downregulated in alkaline conditions |
| orf19.2326 | ESC2 | | -1.6 | | Ortholog(s) have role in chromatin silencing at silent mating-type cassette, double-strand break repair via homologous recombination, intra-S DNA damage checkpoint, mitotic sister chromatid cohesion and nucleus localization |
| orf19.2326 | RAD52**^¶^** | | -1.2 | | Required for homologous DNA recombination, repair of UV- or MMS-damaged DNA, telomere length, UV-induced LOH; constitutive expression, MMS-induced; weakly complements *S. cerevisiae*  rad52 mutant; slow growth, increased white-to-opaque switch |
| orf19.7119 | RAD3**^¶^** | | -1.2 | | Ortholog of *S. cerevisiae*  RAD3; has helicase activity on a duplex DNA substrate. Involved in nucleotide-excision repair, DNA duplex unwinding, DNA incision, phosphorylation of RNA polymerase II C-terminal domain, regulation of mitotic recombination, regulation of transposition, RNA-mediated transcription from RNA polymerase II promoter |
| **Mitochondria** | | | | | |
| orf19.3057 | ILM1 | | -1.8 | | Ortholog(s) have role in mitochondrial genome maintenance and endoplasmic reticulum localization |
| orf19.873.1 | COX6 | | -1.5 | | Putative cytochrome c oxidase; flucytosine induced |
| orf19.3283 | OAR1 | | -3.4 | | Ortholog(s) have 3-oxoacyl-[acyl-carrier-protein] reductase (NADPH) activity, role in aerobic respiration, fatty acid metabolic process and mitochondrion localization |
| orf19.522 | PIM1 | | -1.6 | | Ortholog(s) have ATP-dependent peptidase activity, role in chaperone-mediated protein complex assembly, misfolded or incompletely synthesized protein catabolic process and mitochondrial matrix localization |
| orf19.1267.1 | ISD11 | | -1.5 | | Ortholog(s) have enzyme activator activity, role in iron-sulfur cluster assembly and extrinsic to mitochondrial inner membrane, mitochondrial matrix localization |
| orf19.2192 | GDH2 | | -1.8 | | Putative NAD-specific glutamate dehydrogenase; fungal-specific (no human or murine homolog); transcription is regulated by Nrg1p, Mig1p, Tup1p, and Gcn4p; stationary phase enriched protein; biofilm-induced |
| orf19.6061 | FMP30 | | -1.5 | | Ortholog(s) have role in N-acylethanolamine metabolic process, N-acylphosphatidylethanolamine metabolic process and integral to mitochondrial inner membrane localization |
| orf19.2852 | MRP21 | | -1.5 | | Ortholog(s) have structural constituent of ribosome activity, role in mitochondrial translational initiation and mitochondrial small ribosomal subunit localization |
| orf19.7361 | SEN54 | | -2.4 | | Ortholog(s) have tRNA-intron endonuclease activity, role in tRNA-type intron splice site recognition and cleavage and mitochondrial outer membrane, tRNA-intron endonuclease complex localization |
| orf19.5368 | VMS1 | | -2.2 | | Ortholog(s) have role in ER-associated protein catabolic process, mitochondria-associated protein catabolic process and cytosol, endoplasmic reticulum membrane, mitochondrion localization |
| orf19.7245 | SLM3 | | -1.5 | | Ortholog(s) have tRNA (5-methylaminomethyl-2-thiouridylate)-methyltransferase activity, role in mitochondrial tRNAthio-modification and mitochondrion localization |
| orf19.7187 | MAM33 | | -1.6 | | Putative mitochondrial acidic matrix protein; regulated by Ssn6p; protein present in exponential and stationary growth phase yeast cultures |
| orf19.3929 | AIM25 | | -1.8 | | Ortholog(s) have mitochondrion localization |
| orf19.3040 | EHT1 | | -2.5 | | Protein similar to *S. cerevisiae*  Eht1p; transcription is induced in response to alpha pheromone in SpiderM medium; regulated by Sef1p-, Sfu1p-, and Hap43p |
| orf19.5705 | NAM2 | | -2.1 | | Putative mitochondrial leucyl-tRNAsynthetase |
| orf19.4396 | FCJ1 | | -1.7 | | Ortholog(s) have role in cristae formation, protein import into mitochondrial intermembrane space and integral to mitochondrial inner membrane, mitochondrial crista, mitochondrial crista junction localization |
| orf19.1794 | PET54 | | -1.5 | | Ortholog(s) have RNA binding, translation regulator activity and role in Group I intron splicing, mitochondrial respiratory chain complex IV biogenesis, positive regulation of mitochondrial translation |
| orf19.2384 | MTG1 | | -1.5 | | Ortholog(s) have role in mitochondrial translation and mitochondrial inner membrane localization |
| orf19.522 | PIM1 | | -1.6 | | Ortholog(s) have ATP-dependent peptidase activity, role in chaperone-mediated protein complex assembly, misfolded or incompletely synthesized protein catabolic process and mitochondrial matrix localization |
| orf19.6873.1 | KTI11 | | -1.8 | | Ortholog(s) have electron carrier activity, iron ion binding, zinc ion binding activity, role in peptidyl-diphthamide biosynthetic process from peptidyl-histidine, tRNA wobble uridine modification and cytosol, nucleus localization |
| orf19.639.1 | MRPL44 | | -1.8 | | Ortholog(s) have structural constituent of ribosome activity and mitochondrial large ribosomal subunit localization |
| **Oxidative Stress** | | | | | |
| orf19.7023 | IOC4 | | -1.8 | | Predicted ORF in Assemblies 19, 20 and 21; oxidative stress-induced via Cap1p |
| orf19.2891 | AFG1 | | -1.9 | | Ortholog(s) have role in cellular response to oxidative stress, misfolded or incompletely synthesized protein catabolic process, protein import into peroxisome matrix and mitochondrial inner membrane localization |
| orf19.4510 | IFA4 | | -3.2 | | Predicted ORF in Assemblies 19, 20 and 21; oxidative stress-induced via Cap1p |
| orf19.5816 | EBP7 | | -1.6 | | Putative NADPH oxidoreductase; mutation confers hypersensitivity to toxic ergosterol analog; oxidative stress-induced via Cap1p |
| **Transcription** | | | | | |
| orf19.2998 | TSR2 | | -2.9 | | Putative protein with a predicted role in pre-rRNA processing; decreased expression in response to prostaglandins |
| orf19.7154 | UTP18 | | -2.6 | | Putative U3 snoRNA-associated protein; Hap43p-induced gene; downregulated during core stress response; physically interacts with TAP-tagged Nop1p |
| orf19.2385 | KTI12 | | -2.8 | | Protein similar to *S. cerevisiae*  Kti12p, which associates with Elongator complex; has a role in resistance to killer toxin; predicted Kex2p substrate; Hap43p-induced gene |
| orf19.53 |  | | -1.5 | | Ortholog(s) have role in generation of catalytic spliceosome for first transesterification step, nuclear mRNA cis splicing, via spliceosome and commitment complex, cytosol localization |
| orf19.6903 | RPC37 | | -1.9 | | Ortholog(s) have DNA-directed RNA polymerase activity, role in tRNA transcription from RNA polymerase III promoter and DNA-directed RNA polymerase III complex, cytosol localization |
| orf19.7546 | RRP3 | | -2.2 | | Ortholog(s) have RNA-dependent ATPase activity, role in maturation of SSU-rRNA from tricistronicrRNA transcript (SSU-rRNA, 5.8S rRNA, LSU-rRNA) and cytosol, nucleus localization |
| orf19.5530 | NAB3 | | -2.6 | | Putative nuclear polyadenylated RNA-binding protein; flucytosine repressed |
| orf19.4844 | AIR2 | | -6.8 | | Ortholog(s) have RNA binding, polynucleotide adenylyltransferase activity, protein binding, bridging activity |
| orf19.2111 | TFG2 | | -1.6 | | Ortholog(s) have core RNA polymerase II binding transcription factor activity and role in transcription elongation from RNA polymerase II promoter, transcriptional start site selection at RNA polymerase II promoter |
| orf19.4649 | ZCF27 | | -2.2 | | Putative transcription factor with zinc cluster DNA-binding motif |
| orf19.1304 | RRP4 | | -1.5 | | Ortholog(s) have role in U4 snRNA 3'-end processing, exonucleolytic trimming to generate mature 3'-end of 5.8S rRNAfrom tricistronicrRNA transcript (SSU-rRNA, 5.8S rRNA and LSU-rRNA), more |
| orf19.3912 | GLN3 | | -1.5 | | GATA transcription factor involved in regulation of filamentous growth induced by nitrogen starvation; regulates transcription of Mep2p ammonium permease; regulated by Gcn2p and Gcn4p; mRNA binds to She3p |
| orf19.2528 | BDP1 | | -2.4 | | Putative RNA polymerase III transcription factor (TFIIIB) subunit; flucytosine repressed |
| orf19.6903 | RPC37 | | -1.9 | | Ortholog(s) have DNA-directed RNA polymerase activity, role in tRNA transcription from RNA polymerase III promoter and DNA-directed RNA polymerase III complex, cytosol localization |
| **Translation** | | | | | |
| orf19.1280 | SUI1 | | -1.8 | | Putative translation initiation factor; flucytosine induced; genes encoding ribosomal subunits, translation factors, and tRNAsynthetases are downregulated upon phagocytosis by murine macrophage |
| orf19.6175 | FCF2 | | -2.7 | | Putative 35S rRNA processing protein; Hap43p-induced gene; decreased expression in response to prostaglandins |
| orf19.6584 | PRT1 | | -2.4 | | Putative translation initiation factor eIF3; mutation confers hypersensitivity to roridin A, verrucarin A; genes encoding ribosomal subunits, translation factors, tRNAsynthetases are downregulated upon phagocytosis by murine macrophages |
| orf19.3798 | TRM8 | | -1.7 | | Ortholog(s) have tRNA (guanine-N7-)-methyltransferase activity, role in tRNA methylation and nucleolus, tRNAmethyltransferase complex localization |
| **Translocation and transport** | | | | | |
| orf19.2786 | APL3 | | -1.5 | | Has domain(s) with predicted protein transporter activity, role in intracellular protein transport, vesicle-mediated transport and clathrin adaptor complex localization |
| orf19.4335 | TNA1 | | -2.8 | | Putative nicotinic acid transporter; fungal-specific (no human or murine homolog); detected at germ tube plasma membrane by mass spectrometry; transcriptionally induced upon phagocytosis by macrophage |
| orf19.6624 | GYP6 | | -2.3 | | Ortholog(s) have GTPase activator activity and clathrin-coated vesicle, endosome localization |
| orf19.6551 | GOS1 | | -4.2 | | Ortholog(s) have SNAP receptor activity, role in Golgi vesicle transport, vesicle fusion and Golgi medial cisterna, SNARE complex localization |
| orf19.473 | TPO4 | | -3.3 | | Putative sperimidine transporter; fungal-specific (no human or murine homolog) |
| orf19.7670 | VNX1 | | -2.4 | | Putative Ca2+/H+ antiporter; oral infection upregulated gene; mutants have reduced capacity to damage oral epithelial cells |
| orf19.3746 | IFC1 | | -2.1 | | Oligopeptide transporter; induced upon phagocytosis by macrophage; macrophage/pseudohyphal-repressed after 16h; fluconazole-induced; virulence-group-correlated expression; fungal-specific (no mammalian homolog); Hap43p-repressed |
| orf19.4118 | CNT | | -3.5 | | CNT family H(+)/nucleoside symporter; transports adenosine, uridine, inosine, guanosine, tubercidin; variant alleles encode high/low-affinity isoforms; S or G at residue 328 affects specificity; late-stage biofilm-induced |
| orf19.5539 | USE1 | | -2.9 | | Ortholog(s) have role in retrograde vesicle-mediated transport, Golgi to ER and SNARE complex, integral to cytosolic side of endoplasmic reticulum membrane localization |
| orf19.3226 | NPC2 | | -3.4 | | Ortholog(s) have role in intracellular sterol transport and fungal-type vacuole lumen localization |
| orf19.5276 | THP1 | | -2.0 | | Putative nuclear pore-associated protein; Hap43p-induced gene; induced upon low-level peroxide stress; possibly an essential gene, disruptants not obtained by UAU1 method |
| orf19.2991 | HOL1 | | -1.5 | | Putative MFS transporter; transcription regulated by Nrg1p; macrophage/pseudohyphal-repressed; transcription induced by alpha pheromone in SpiderM medium; possibly an essential gene, disruptants not obtained by UAU1 method |
| orf19.2974 | YKT6 | | -1.8 | | Putative protein of the vacuolar SNARE complex with a predicted role in vacuolar fusion |
| orf19.3573 | PEX6 | | -1.7 | | Ortholog(s) have ATPase activity, protein heterodimerization activity and role in protein import into peroxisome matrix, receptor recycling, replicative cell aging |
| orf19.2895 | VMA8 | | -1.6 | | Protein similar to the *S. cerevisiae*  Vma8p subunit of vacuolar H+-ATPase; transcription is regulated by Nrg1p and Mig1p; transcription is increased in populations of cells exposed to fluconazole over multiple generations |
| orf19.6005 | HGT5 | | -1.8 | | Putative glucose transporter of the major facilitator superfamily; the C. *albicans* glucose transporter family comprises 20 members; 12 probable membrane-spanning segments, extended N terminus; expressed in rich medium; Hap43p-repressed |
| orf19.4059 | YHC3 | | -1.8 | | Ortholog(s) have role in actin cortical patch localization, arginine transport, cellular protein localization, cellular response to heat, endocytosis and late endosome to vacuole transport, more |
| orf19.2055 | NPL6 | | -2.1 | | Putative nuclear protein localization factor; fungal-specific (no human or murine homolog; Hap43p-induced gene |
| orf19.5340 | GYP5 | | -1.6 | | Ortholog(s) have RabGTPase activator activity and role in ER to Golgi vesicle-mediated transport |
| orf19.3314 | TRS20 | | -2.2 | | Ortholog(s) have role in ER to Golgi vesicle-mediated transport and TRAPP complex, cytosol, nucleus localization |
| orf19.7205 | DUR7 | | -1.5 | | Putative urea transporter; fungal-specific (no human or murine homolog) |
| orf19.5962 | HGT4 | | -1.98 | | Glucose and galactose sensor; fermentation, filamentation, virulence roles; in 20-member C. *albicans* glucose transporter family, extended C terminus; high-glucose repressed; induced in macrophage, repressed in biofilm |
| orf19.6590 | VMA22 | | -1.5 | | Ortholog(s) have unfolded protein binding activity, role in vacuolar acidification, vacuolar proton-transporting V-type ATPase complex assembly and extrinsic to endoplasmic reticulum membrane localization |
| orf19.7148 | TPO2 | | -1.7 | | Putative polyamine transport protein; fungal-specific (no human or murine homolog) |
| orf19.4593 | RGA2 | | -1.6 | | Putative GTPase-activating protein (GAP) for Rho-type GTPase Cdc42p; involved in cell signaling pathways controlling cell polarity; similar to *S. cerevisiae*  Rga2p; induced upon low-level peroxide stress; late-stage biofilm-induced |
| orf19.3931 | SFC1 | | -1.6 | | Putative succinate-fumarate transporter; gene is involved in repression of growth on sorbose; alkaline upregulated; transcription is upregulated in both intermediate and mature biofilms |
| orf19.880 | SFB3 | | -1.7 | | Ortholog(s) have role in cargo loading into COPII-coated vesicle and COPII vesicle coat, cytosol, mating projection tip localization |
| orf19.5825 | NCB2 | | -1.98 | | Beta subunit of NC2, heterodimeric regulator of transcription; activates CDR1 transcription; possibly an essential gene, disruptants not obtained by UAU1 method; conditional mutants show decreased susceptibility to azoles |
| orf19.1866 | VMA10 | | -1.8 | | Ortholog(s) have cytosol, nucleus, vacuolar proton-transporting V-type ATPase, V1 domain localization; GlcNAc-induced protein |
| orf19.5753 | HGT10 | | -2.2 | | Glycerol permease involved in glycerol uptake; member of the major facilitator superfamily; induced by osmotic stress, at low glucose in rich media, during cell wall regeneration; 12 membrane spans; Hap43p-induced gene |
| orf19.3669 | SHA3 | | -1.5 | | Putative serine/threonine kinase involved in glucose transport; transposon mutation affects filamentous growth; fluconazole-induced; ketoconazole-repressed; induced in by alpha pheromone; possibly essential (UAU1 method); biofilm-induced |
| **Cellular metabolic processes** | | | | | |
| orf19.6659 | GAP6 | | -4.1 | | Broad-specificity amino acid permease; Plc1p-regulated; Gcn4p-regulated; fungal-specific (no human or murine homolog) |
| orf19.5112 | TKL1 | | -4.2 | | Putative transketolase; localizes to surface of yeast-form cells, but not hyphae; soluble protein in hyphae; transcription regulated by Nrg1p, Mig1p, and Tup1p; antigenic in human or murine infection; possibly essential (by UAU1 method) |
| orf19.4866 | CPP1 | | -2.8 | | VH1 family MAPK phosphatase; regulates Cst20p-Hst7p-Cek1p-Cph1p hyphal growth pathway; represses yeast to hyphal switch; required for virulence in mouse systemic infection; more mRNA in yeast than hyphae; pheromone-induced |
| orf19.7179 | SSQ1 | | -3.3 | | Ortholog(s) have role in intracellular sequestering of iron ion, iron-sulfur cluster assembly, protein maturation and mitochondrial matrix localization |
| orf19.6987 | DNM1 | | -3.1 | | Putative dynamin-related GTPase; transcription is upregulated in an RHE model of oral candidiasis; transcription is regulated by Nrg1p, Mig1p, and Tup1p |
| orf19.5184 | SRM1 | | -1.8 | | Ortholog(s) have Ran guanyl-nucleotide exchange factor activity, signal transducer activity |
| orf19.2504 | BMS1 | | -2.4 | | Putative GTPase; Hap43p-induced gene; mutation confers resistance to 5-fluorocytosine (5-FC); flucytosine induced; decreased expression in response to prostaglandins |
| orf19.3934 | CAR1 | | -1.9 | | Arginase involved in arginine catabolism; transcription regulated by Nrg1p, Mig1p, and Tup1p; colony morphology-related regulation by Ssn6p; alkaline upregulated; protein decreased in stationary phase; biofilm-induced; sumoylation target |
| orf19.5450 | ETR1 | | -2.0 | | Putative 2-enoyl thioester reductase; protein present in exponential and stationary growth phase yeast cultures |
| orf19.4738 | SLP1 | | -2.4 | | Ortholog(s) have role in protein folding in endoplasmic reticulum |
| orf19.5337 | UBC15 | | -1.5 | | Putative E2 ubiquitin-conjugating enzyme |
| orf19.1377 | IPK2 | | -1.6 | | Putative inositol polyphosphate multikinase; moderately induced at 42 deg |
| orf19.2614 | RSR1 | | -2.3 | | RAS-related protein; GTP/GDP cycling required for wild-type polar bud site selection, hyphal growth guidance; role in systemic virulence in mouse; geranylgeranylation predicted; suppresses *S. cerevisiae*  cdc24-4 mutant heat sensitivity |
| orf19.3260 | CAB3 | | -1.8 | | Ortholog(s) have phosphopantothenoylcysteine decarboxylase activity, role in coenzyme A biosynthetic process, response to salt stress and phosphopantothenoylcysteine decarboxylase complex localization |
| orf19.3956 | HER2 | | -2.6 | | Ortholog(s) have glutaminyl-tRNA synthase (glutamine-hydrolyzing) activity and role in endoplasmic reticulum organization, glutaminyl-tRNAGln biosynthesis via transamidation |
| orf19.1977 | URA4 | | -3.4 | | Dihydroorotase; protein present in exponential and stationary growth phase yeast cultures |
| orf19.2529 | GPI15 | | -1.8 | | Predicted ORF in Assemblies 19, 20 and 21; shares similarity with human Pig-H, which is involved in glycosylphosphatidylinositol assembly |
| orf19.5864 | URK1 | | -1.8 | | Protein similar to uridine kinase; transcription is downregulated in response to treatment with ciclopiroxolamine |
| orf19.5833 | UFD1 | | -1.6 | | Ortholog(s) have polyubiquitin binding activity and role in ER-associated misfolded protein catabolic process, cytoplasm-associated proteasomal ubiquitin-dependent protein catabolic process, retrograde protein transport, ER to cytosol |
| orf19.1110 | THI80 | | -1.7 | | Thiamine pyrophosphokinase, phosphorylates thiamine to produce the coenzyme thiamine pyrophosphate (thiamine diphosphate); active as a homodimer |
| orf19.641 | ERJ5 | | -2.1 | | Ortholog(s) have role in protein folding and endoplasmic reticulum localization |
| orf19.5641 | CAR2 | | -1.6 | | Ornithine aminotransferase; role in arginine metabolism; alkaline upregulated; mutation confers hypersensitivity to toxic ergosterol analog and to amphotericin B; present in exponential and stationary phase yeast cultures; biofilm-induced |
| orf19.1699 | BZZ1 | | -3.4 | | Protein similar to *S. cerevisiae*  Bzz1p, which is an SH3 domain protein involved in the regulation of actin polymerization |
| orf19.4233 | THR4 | | -3.0 | | Putative threonine synthase; protein present in exponential and stationary growth phase yeast cultures; Gcn4p-regulated; sumoylation target |
| orf19.7546 | RRP3 | | -2.2 | | Ortholog(s) have RNA-dependent ATPase activity, role in maturation of SSU-rRNA from tricistronicrRNA transcript (SSU-rRNA, 5.8S rRNA, LSU-rRNA) and cytosol, nucleus localization |
| **Cell communication** | | | | | |
| orf19.1914 | FAV3 | | -2.5 | | Putative alpha-1,6-mannanase; induced by mating factor in MTLa/MTLa opaque cells |
| **Virulence** | | | | | |
| orf19.2151 | NAG6 | | -1.9 | | Protein required for wild-type mouse virulence and wild-type cycloheximide resistance; putative GTP-binding motif; similar to *S. cerevisiae*  Yor165Wp; in gene cluster that encodes enzymes of GlcNAc catabolism; no human or murine homolog |
| orf19.988 | YGR149W | | -2.3 | | Oral infection upregulated gene; transcription upregulated in strains from HIV+ patients with oral candidiasis; mutants have reduced capacity to damage oral epithelial cells; possibly transcriptionally regulated upon hyphal formation |
| **Uncharacterized genes** | | | | | |
| orf19.7579 | | FGR34 | | -1.8 | Protein lacking an ortholog in *S. cerevisiae* ; transposon mutation affects filamentous growth |
| orf19.292 | |  | | -3.0 | Ortholog of *C. parapsilosis* CDC317 : CPAR2_102390, *Candida* *tenuis* NRRL Y-1498 : CANTEDRAFT_133198, Lodderomyceselongisporus NRLL YB-4239 : LELG_02116 and *Candida* *dubliniensis* CD36 : CD36_82960 |
| orf19.3722 | | FAP1 | | -1.8 | Ortholog(s) have cytoplasm localization |
| orf19.5710 | | NUP1 | | -1.7 | Putative protein of unknown function; mRNA binds to She3p |
| orf19.442 | |  | | -1.6 | ORF Predicted by Annotation Working Group; overlaps RPT1/orf19.441 |
| orf19.3517 | | YOR223W | | -4.2 | Ortholog(s) have endoplasmic reticulum, fungal-type vacuole lumen localization |
| orf19.6276 | |  | | -1.8 | Ortholog of *C. parapsilosis* CDC317 : CPAR2_803870, *Candida* *tenuis* NRRL Y-1498 : CANTEDRAFT_112418, Debaryomyceshansenii CBS767 : DEHA2G15246g and *Candida* *dubliniensis* CD36 : CD36_05930 |
| orf19.4123 | | THO2 | | -3.4 | Putative THO complex subunit; possibly an essential gene, disruptants not obtained by UAU1 method |
| orf19.2074 | |  | | -2.0 | Ortholog of *Candida* *albicans* WO-1 : CAWG_03833 |
| orf19.4583 | |  | | -1.5 | Predicted ORF in Assemblies 19, 20 and 21; possibly an essential gene, disruptants not obtained by UAU1 method |
| orf19.5763 | |  | | -1.6 | Has domain(s) with predicted nucleotide binding, oxidoreductase activity and role in oxidation-reduction process |
| orf19.2018.2 | |  | | -2.1 | Ortholog of *Candida tropicalis* MYA-404:CTRG_04298 |
| orf19.3439 | |  | | -3.0 | Predicted ORF in Assemblies 19, 20 and 21; greater mRNA abundance observed in a cyr1 homozygous null mutant than in wild type |
| orf19.5064.1 | |  | | -3.7 | Ortholog of *C. parapsilosis* CDC317 : CPAR2_208450, *Candida* *tenuis* NRRL Y-1498 : CANTEDRAFT_116488, Debaryomyceshansenii CBS767 : DEHA2F26114g and *Candida* *dubliniensis* CD36 : CD36_07450 |
| orf19.2584 | | OPT9 | | -2.1 | Probable pseudogene similar to fragments of OPT1 oligopeptide transporter gene; decreased expression in hyphae compared to yeast-form cells; transcriptionally induced upon phagocytosis by macrophage |
| orf19.5030 | | DOS2 | | -1.5 | Predicted ORF in Assemblies 19, 20 and 21; induced during planktonic growth |
| orf19.6470 | | AHP2 | | -2.3 | Putative thiol-specific peroxiredoxin; macrophage-downregulated gene |
| orf19.6678 | |  | | -1.6 | Hap43p-repressed gene |
| orf19.675.1 | |  | | -1.8 | Putative adhesin-like protein |
| orf19.3461 | |  | | -3.4 | Predicted ORF in Assemblies 19, 20 and 21; oxidative stress-induced via Cap1p; transcription is induced in response to alpha pheromone in SpiderM medium |
| orf19.4510 | | IFA4 | | -3.2 | Predicted ORF in Assemblies 19, 20 and 21; oxidative stress-induced via Cap1p |
| orf19.441 | | RPT1 | | -3.3 | Putative 26S proteasome regulatory subunit 7; Hap43p-repressed gene; regulated by Gcn2p and Gcn4p; overlaps orf19.442 |
| orf19.1774 | |  | | -1.5 | Predicted ORF in Assemblies 19, 20 and 21; transcription is upregulated in an RHE model of oral candidiasis; virulence-group-correlated expression |
| orf19.2195 | |  | | -2.3 | Ortholog of *Candida* *albicans* WO-1 : CAWG_05881 |
| orf19.2826 | |  | | -1.5 | Ortholog of *C. parapsilosis* CDC317 : CPAR2_801140, *Candida* *tenuis* NRRL Y-1498 : CANTEDRAFT_129982, Debaryomyceshansenii CBS767 : DEHA2E09526g and *Candida* *dubliniensis* CD36 : CD36_27680 |
| orf19.4057 | |  | | -1.6 | Ortholog of *Candida* *dubliniensis* CD36 : CD36_04885 and *Candida* *albicans* WO-1 : CAWG_00885 |
| orf19.7322 | | YPL225W | | -1.5 | Ortholog(s) have cytosol, nucleus, ribosome localization |
| orf19.3325 | | GLG2 | | -1.5 | Putative glycogen synthesis initiator; regulated by Efg1p and Efh1p; Hog1p-downregulated; shows colony morphology-related gene regulation by Ssn6p; increased expression in response to prostaglandins; biofilm-induced |
| orf19.201 | | MCM7 | | -1.7 | Predicted ORF in Assemblies 19, 20 and 21; unmerged from orf19.202 in a revision of Assembly 21 |
| orf19.1323 | | APL6 | | -1.6 | Phosphorylated protein of unknown function; Hap43p-repressed gene |
| orf19.955 | |  | | -1.5 | Ortholog of *C. parapsilosis* CDC317 : CPAR2_304150, *Candida* *tenuis* NRRL Y-1498 : CANTEDRAFT_135060, Debaryomyceshansenii CBS767 : DEHA2B07810g and *Candida* *dubliniensis* CD36 : CD36_50370 |
| orf19.6597 | | YLR287C | | -2.3 | Ortholog(s) have cytoplasm localization |
| orf19.3826 | | SKG1 | | -2.2 | Predicted ORF in Assemblies 19, 20 and 21; Plc1p-regulated |
| orf19.4407 | |  | | -2.3 | Hap43p-repressed gene |
| orf19.1834 | |  | | -1.6 | Ortholog of *C. parapsilosis* CDC317 : CPAR2_212580, Debaryomyceshansenii CBS767 : DEHA2G11770g, *Candida* *dubliniensis* CD36 : CD36_09960 and Pichia stipitisPignal : PICST_51041 |
| orf19.5020 | |  | | -4.8 | Hap43p-induced gene |
| orf19.2472 | |  | | -2.0 | Ortholog(s) have nucleus localization |
| orf19.3045 | |  | | -1.5 | Predicted ORF in Assemblies 19, 20 and 21; virulence-group-correlated expression |
| orf19.5720 | |  | | -2.3 | Predicted membrane transporter, member of the monocarboxylate porter (MCP) family, major facilitator superfamily (MFS); ketoconazole or caspofungin repressed |
| orf19.3780 | |  | | -2.4 | Protein likely to be essential for growth, based on an insertional mutagenesis strategy |
| orf19.3719 | |  | | -1.8 | Ortholog of *Candida* *dubliniensis* CD36 : CD36_27410 and *Candida* *albicans* WO-1 : CAWG_01588 |
| orf19.7666 | |  | | -1.5 | Predicted membrane transporter, member of the anion:cation symporter (ACS) family, major facilitator superfamily (MFS) |
| orf19.609 | |  | | -1.6 | Predicted ORF in Assemblies 19, 20 and 21; transcription is specific to white cell type |
| orf19.2304 | |  | | -2.1 | Protein similar to *S. cerevisiae*  Gvp36p; transposon mutation affects filamentous growth |
| orf19.27 | |  | | -1.5 | Ortholog of *C. parapsilosis* CDC317 : CPAR2_104250, *Candida* *tenuis* NRRL Y-1498 : CANTEDRAFT_115100, Debaryomyceshansenii CBS767 : DEHA2E10450g and *Candida* *dubliniensis* CD36 : CD36_20860 |
| orf19.3679 | | YNL200C | | -1.6 | Putative protein of unknown function; stationary phase enriched protein |
| orf19.1668 | | SCM3 | | -2.4 | Protein of unknown function; expression downregulated in an ssr1 null mutant |
| orf19.1642 | | LOC1 | | -1.6 | Hap43p-induced gene; ortholog of *S. cerevisiae*  LOC1, a nuclear protein involved in asymmetric localization of ASH1 mRNA in *S. cerevisiae* |
| orf19.909.1 | |  | | -2.2 | Predicted ORF in Assembly 20 and 21; gene has intron |

¥: Gene names and ORF numbers according to the *Candida* genomic database.

*: The average expression ratio (Log_2_ fold changes) from three independent experiments is shown. Negative numbers indicate downregulation.

¶: These genes were partially downregulated (<1.5) but are included considering their significance to the present study.
