# Supplementary material for: Anticandidal Effect and Mechanisms of Monoterpenoid, Perillyl Alcohol against Candida albicans
Source: PLoS One. 2016 Sep 14;11(9):e0162465. doi: 10.1371/journal.pone.0162465 (PMC5023166; doi:10.1371/journal.pone.0162465)
Supplement: S3 Table — (DOCX) [file pone.0162465.s005.docx]

**S3 Table. List of upregulated genes in response to PA.**

| **ORF ID^¥^** | **Standard gene name^¥^** | | | | | **Fold change^*^(Log_2_)** | **Description** |
| --- | --- | --- | --- | --- | --- | --- | --- |
| **Plasma membrane genes** | | | | | | | |
| orf19.4699 | TGL3 | | | | | 1.5 | Putative phospholipase of patatin family; similar to *S. cerevisiae*  Tgl3p; predicted Kex2p substrate |
| orf19.656 | DPP1 | | | | | 2.4 | Putative diacylglycerol pyrophosphate phosphatase of diacylglycerol production for phospholipid biosynthesis; downregulation correlates with clinical development of fluconazole resistance |
| **Yeast to Hypha transition** | | | | | | | |
| orf19.6598 | WAL1 | | 3.2 | | | | Protein required for hyphal growth and for wild-type cell morphology, polarized budding, endocytosis, vacuole morphology; similar to Wiskott-Aldrich syndrome protein; localizes to cortical actin patches and hyphal tips |
| **Calcineurin signaling** | | | | | | | |
| orf19.3329 | LCB3 | | 2.01 | | | | Ortholog(s) have sphingosine-1-phosphate phosphatase activity, role in calcium-mediated signaling and endoplasmic reticulum localization |
| orf19.405 | VCX1 | | 2.05 | | | | Putative H+/Ca2+ antiporter; fungal-specific (no human or murine homolog) |
| orf19.7089 | PMR1 | | 1.5 | | | | Putative secretory pathway P-type Ca2+/Mn2+-ATPase; required for protein glycosylation and cell wall maintenance; required for hyphal tip oscillation in semisolid substrate; putative ortholog of *S. cerevisiae*  PMR1 |
| **Cell wall** | | | | | | | |
| orf19.4765 | PGA6 | | 2.5 | | | | Putative GPI-anchored cell wall adhesin-like protein; transcriptionally induced under high iron conditions; upregulated upon Als2p depletion; mRNA binds to She3p and is localized to hyphal tips |
| orf19.7084 | DFI1 | | 2.3 | | | | Cell-surface associated glycoprotein; promotes activation of Cek1p in a matrix-dependent manner; N-glycosylated |
| orf19.2990 | XOG1 | | 3.1 | | | | Exo-1,3-beta-glucanase; 5 glycosyl hydrolase family member; affects sensitivity to chitin and glucan synthesis inhibitors; not required for yeast-to-hyphal transition or for virulence in mouse systemic infection; Hap43p-induced gene |
| orf19.7676 | XYL2 | | 1.7 | | | | Putative D-xylulose reductase; immunogenic in mice; soluble protein in hyphae; induced by caspofungin, fluconazole, Hog1p and during cell wall regeneration; Mnl1p-induced in weak acid stress; stationary phase enriched; biofilm-induced |
| orf19.1665 | MNT1 | | 1.8 | | | | Alpha-1,2-mannosyl transferase; adds second mannose during cell-wall mannoprotein biosynthesis; required for wild-type virulence and adherence to epithelial cells; predicted type II Golgi membrane protein; fungal-specific; Hap43p-induced |
| **Cell cycle arrest/Cytoskeleton organisation/DNA replication** | | | | | | | |
| orf19.4867 | SWE1 | | 1.8 | | | | Putative protein kinase with a role in control of growth and morphogenesis, required for full virulence; mutant cells are small, rounded, and sometimes binucleate; not required for filamentous growth; mutant is hypersensitive to caspofungin |
| orf19.513 |  | | 1.6 | | | | Protein with a DNA-binding domain, similar to *S. cerevisiae*  meiosis-specific transcription factor Ndt80p |
| orf19.6049 | SLI15 | | 1.8 | | | | Ortholog(s) have protein kinase activator activity, role in protein phosphorylation, regulation of cytokinesis and chromosome passenger complex, kinetochore microtubule, spindle midzone localization |
| orf19.2070 | RSC58 | | 2.1 | | | | Ortholog(s) have role in G1/S transition of mitotic cell cycle, chromatin remodeling, nucleosome disassembly, transcription elongation from RNA polymerase II promoter and RSC complex localization |
| **Mitochondria** | | | | | | | |
| orf19.3419 | MAE1 | | 1.6 | | | | Malic enzyme, mitochondrial; transcription regulated by Mig1p and Tup1p; shows colony morphology-related gene regulation by Ssn6p; Hap43p-repressed gene |
| orf19.5000 | CYB2 | | 2.4 | | | | Putative cytochrome b2 precursor protein; transcriptionally regulated by iron; expression greater in high iron; alkaline downregulated; shows colony morphology-related gene regulation by Ssn6p; Hap43p-repressed |
| orf19.5776 | TOM1 | | 1.9 | | | | Putative E3 ubiquitin ligase; transcription is regulated by Nrg1p and Mig1p |
| **Transcription** | | | | | | | |
| orf19.1718 | ZCF8 | | 1.5 | | | | Predicted zinc-finger protein; decreased transcription is observed upon fluphenazine treatment; required for yeast cell adherence to silicone substrate |
| orf19.6314 | RPB8 | | 1.7 | | | | Putative subunit of RNA polymerases I, II, and III; regulated by Gcn4p; repressed in response to amino acid starvation (3-aminotriazole treatment); heterozygous null mutant exhibits resistance to parnafungin |
| orf19.1499 | CTF1 | | 2.0 | | | | Putative zinc-finger transcription factor, similar to *A. nidulans* FarA and FarB; activates genes required for fatty acid degradation; induced by oleate; null mutant displays carbon source utilization defects and slightly reduced virulence |
| orf19.2748 | ARG83 | | 1.7 | | | | Zinc-finger protein; clade-associated gene expression; null mutant shows abnormal regulation of invasive colony growth and is unable to utilize proline as a nitrogen source; late-stage biofilm-induced |
| orf19.6314 | RPB8 | | 1.7 | | | | Putative subunit of RNA polymerases I, II, and III; regulated by Gcn4p; repressed in response to amino acid starvation (3-aminotriazole treatment); heterozygous null mutant exhibits resistance to parnafungin |
| **Translation** | | | | | | | |
| orf19.5351 | TIF11 | | 2.2 | | | | Translation initiation factor eIF1a; possibly transcriptionally regulated upon hyphal formation; genes encoding ribosomal subunits, translation factors, and tRNAsynthetases are downregulated upon phagocytosis by murine macrophage |
| orf19.4279 | MNN1 | | 3.7 | | | | Putative alpha-1,3-mannosyltransferase, a component of mannosyltransferase complex; transcription negatively regulated by Rim101p; transcription elevated in chk1 and nik1 null mutants, but not in sln1 null mutant; biofilm-induced gene |
| orf19.4900 | MNT2 | | 2.8 | | | | Ortholog(s) have alpha-1,3-mannosyltransferase activity and role in protein O-linked glycosylation |
| orf19.3341 | YDR341C | | 2.1 | | | | Putative tRNA-Argsynthetase; essential; genes encoding ribosomal subunits, translation factors, and tRNAsynthetases are downregulated upon phagocytosis by murine macrophage; downregulated by growth in the mouse cecum |
| orf19.4283 |  | | 2.9 | | | | Ortholog(s) have role in translational initiation and cytosol, eukaryotic 43S preinitiation complex, eukaryotic translation initiation factor 3 complex, eIF3e, eukaryotic translation initiation factor 3 complex, eIF3m, nucleus localization |
| orf19.261 | SEC59 | | 1.9 | | | | Ortholog(s) have dolichol kinase activity, role in protein glycosylation and endoplasmic reticulum membrane localization |
| **Translocation and transport** | | | | | | | |
| orf19.30 | SPF1 | | | 1.8 | | | Putative calcium-transporting ATPase, involved in control of calcium homeostasis, response to ER stress, hyphal growth, biofilm formation and virulence |
| orf19.3366 | CSH3 | | | 2.4 | | | Functional homolog of *S. cerevisiae*  Shr3p, which is a chaperone specific for amino acid permeases; localized to ER; required for wild-type amino-acid responsive hyphal growth and for mouse systemic virulence; regulated by Gcn2p and Gcn4p |
| orf19.645.1 | VMA13 | | | 1.5 | | | Has domain(s) with predicted proton-transporting ATPase activity, rotational mechanism activity, role in ATP hydrolysis coupled proton transport and vacuolar proton-transporting V-type ATPase, V1 domain localization |
| orf19.1655.3 | PMP3 | | | 1.5 | | | Ortholog(s) have role in cation transport, regulation of membrane potential and plasma membrane localization |
| orf19.6665 | NUP2 | | | 2.1 | | | Ortholog(s) have nucleocytoplasmic transporter activity |
| **Iron** | | | | | | | |
| orf19.424 | TRP99 | | 1.6 | | | | Putative thioredoxin peroxidase/alkyl hydroperoxide reductase; transcriptionally regulated by iron; expression greater in low iron; regulated by Gcn4p; induced in response to amino acid starvation (3-aminotriazole treatment) |
| orf19.30 | FTR2 | | 1.6 | | | | High-affinity iron permease; probably interacts with ferrous oxidase; regulated by iron level, ciclopiroxolamine, amphotericin B, caspofungin; complements *S. cerevisiae*  ftr1 iron transport defect; Hap43p-repressed |
| **Cellular metabolic processes** | | | | | | | |
| orf19.946 | MET14 | | | 2.3 | | | Putative adenylylsulfate kinase; predicted role in sulfur metabolism; induced upon biofilm formation; possibly adherence-induced; protein present in exponential and stationary growth phase yeast cultures |
| orf19.5159 | DUG3 | | | 1.9 | | | Ortholog(s) have gamma-glutamyltransferase activity, omega peptidase activity, role in glutathione catabolic process and cytoplasm localization |
| orf19.7269 | PAA1 | | | 2.6 | | | Ortholog(s) have aralkylamine N-acetyltransferase activity, diamine N-acetyltransferase activity, role in chromatin organization and cytoplasm localization |
| orf19.271 | ADH4 | | | 2.4 | | | Protein not essential for viability; transcription is increased in an azole-resistant strain overexpressing MDR1; transcription is increased in populations of cells exposed to fluconazole over multiple generations |
| orf19.3396 | HCH1 | | | 1.7 | | | Protein not essential for viability; regulated by Gcn4p; induced in response to amino acid starvation (3-aminotriazole treatment); similar to *S. cerevisiae*  Hch1p, which is a regulator of heat shock protein Hsp90p |
| orf19.1359 | UBX4 | | | 1.5 | | | Ortholog(s) have protein complex binding activity, role in ER-associated protein catabolic process, sporulation resulting in formation of a cellular spore and cytoplasm, nucleus, peripheral to membrane of membrane fraction localization |
|  |  | | |  | | |  |
| orf19.5912 | MAK21 | | | 3.5 | | | Putative 66S pre-ribosomal particle subunit; mutation confers hypersensitivity to tubercidin (7-deazaadenosine) |
| orf19.2124 |  | | | 3.9 | | | Has domain(s) with predicted nucleotide binding, oxidoreductase activity, transferase activity, transferring acyl groups other than amino-acyl groups, zinc ion binding activity and role in oxidation-reduction process |
| orf19.4172 |  | | | 1.5 | | | Has domain(s) with predicted hydrolase activity and role in metabolic process |
| orf19.7394 | GDA1 | | | 1.5 | | | Golgi membrane GDPase, required for wild-type O-mannosylation, not N-glycosylation; required for wild-type hyphal induction, cell wall, and cell surface charge; not required for HeLa cell adherence; functional homolog of *S. cerevisiae*  Gda1p |
| orf19.1448 | APT1 | | | 2.0 | | | Adenine phosphoribosyltransferase; flucytosine induced; repressed by nitric oxide; protein level decreased in stationary phase yeast cultures |
| **Cell communication** | | | | | | | |
| orf19.2795 | LHP1 | | 1.7 | | | | Ortholog(s) have tRNA binding activity, role in regulation of ascospore formation, regulation of conjugation with cellular fusion, tRNA 3'-trailer cleavage and nucleolus, nucleoplasm localization |
| **Uncharacterized genes** | | | | | | | |
| orf19.5299 | | ECM1 | | | 1.9 | | Putative pre-ribosomal factor; decreased mRNA abundance observed in cyr1 homozygous mutant hyphae; induced by heavy metal (cadmium) stress; Hog1p regulated |
| orf19.3600 | |  | | | 1.7 | | Late-stage biofilm-induced gene |
| orf19.6637 | |  | | | 1.9 | | Biofilm- and planktonic growth-induced gene; induced by hypoxia |
| orf19.2177 | |  | | | 1.7 | | Sef1p-, Sfu1p-, and Hap43p-regulated gene; overlaps IFM3/orf19.2176 |
| orf19.5079.1 | |  | | | 1.9 | | Ortholog of *C. parapsilosis* CDC317 : CPAR2_206680, *Candida* *tenuis* NRRL Y-1498 : CANTEDRAFT_104385, Debaryomyceshansenii CBS767 : DEHA2C10934g and *Candida* *dubliniensis*CD36 : CD36_07590 |
| orf19.4674.1 | | CRD2 | | | 3.3 | | Metallothionein; role in adaptation to growth in high copper; basal transcription is cadmium-repressed; regulated by Ssn6p; complements copper sensitivity of an *S. cerevisiae*  cup1 null mutant; regulated by Sef1p-, Sfu1p-, and Hap43p |
| orf19.3678 | |  | | | 2.2 | | Protein not essential for viability |
| orf19.2257 | | YNR021W | | | 1.9 | | Ortholog(s) have endoplasmic reticulum localization |
| orf19.6324 | | VID27 | | | 2.1 | | Protein similar to *S. cerevisiae*  Vid27p; transposon mutation affects filamentous growth; mutation confers hypersensitivity to toxic ergosterol analog; fungal-specific (no human or murine homolog) |
| orf19.5231 | |  | | | 2.0 | | Late-stage biofilm-induced gene |
| orf19.4756 | | YTP1 | | | 1.5 | | Ortholog of *S. cerevisiae*  : YTP1, *C. parapsilosis* CDC317 : CPAR2_801590, *Candida* *tenuis* NRRL Y-1498 : CANTEDRAFT_109732 and Debaryomyceshansenii CBS767 : DEHA2C10384g |
| orf19.6644 | |  | | | 2.1 | | Biofilm-induced gene; transcription detected in high-resolution tiling array experiments |
| orf19.2994.1 | | RPS16A | | | 1.7 | | Putative 40S ribosomal subunit; macrophage/pseudohyphal-induced after 16 h |
| orf19.4847 | |  | | | 2.1 | | ORF Predicted by Annotation Working Group; overlaps orf19.4846 |
| orf19.2819 | |  | | | 1.8 | | Ortholog of *C. parapsilosis* CDC317 : CPAR2_801010, Debaryomyceshansenii CBS767 : DEHA2E09306g, *Candida* *dubliniensis* CD36 : CD36_27570 and Pichia stipitisPignal : PICST_30503 |

¥: Gene names and ORF numbers according to the *Candida* genomic database.

*: The average expression ratio (Log_2_ fold changes) from three independent experiments is shown. Positive numbers indicate upregulation.
